# Potential early clinical stage colorectal cancer diagnosis using a proteomics blood test panel

**DOI:** 10.1186/s12014-019-9255-z

**Published:** 2019-08-28

**Authors:** Seong Beom Ahn, Samridhi Sharma, Abidali Mohamedali, Sadia Mahboob, William J. Redmond, Dana Pascovici, Jemma X. Wu, Thiri Zaw, Subash Adhikari, Vineet Vaibhav, Edouard C. Nice, Mark S. Baker

**Affiliations:** 1 Department of Biomedical Sciences, Faculty of Medicine and Health Sciences, Macquarie University, Level 1, 75 Talavera Road, Sydney, NSW 2109 Australia; 2 Department of Molecular Sciences, Faculty of Science and Engineering, Macquarie University, Sydney, NSW 2109 Australia; 3 Australian Proteome Analysis Facility (APAF), Department of Molecular Sciences, Faculty of Science and Engineering, Macquarie University, Sydney, NSW 2109 Australia; 4 Department of Biochemistry and Molecular Biology, Faculty of Medicine, Nursing and Health Sciences, Monash University, Clayton, VIC 3800 Australia

**Keywords:** Colorectal cancer, SWATH-MS, Plasma protein biomarkers, High abundant protein depletion, Early stage diagnosis, Predictive model

## Abstract

**Background:**

One of the most significant challenges in colorectal cancer (CRC) management is the use of compliant early stage population-based diagnostic tests as adjuncts to confirmatory colonoscopy. Despite the near curative nature of early clinical stage surgical resection, mortality remains unacceptably high—as the majority of patients diagnosed by faecal haemoglobin followed by colonoscopy occur at latter stages. Additionally, current population-based screens reliant on fecal occult blood test (FOBT) have low compliance (~ 40%) and tests suffer low sensitivities. Therefore, blood-based diagnostic tests offer survival benefits from their higher compliance (≥ 97%), if they can at least match the sensitivity and specificity of FOBTs. However, discovery of low abundance plasma biomarkers is difficult due to occupancy of a high percentage of proteomic discovery space by many high abundance plasma proteins (e.g., human serum albumin).

**Methods:**

A combination of high abundance protein ultradepletion (e.g., MARS-14 and an in-house IgY depletion columns) strategies, extensive peptide fractionation methods (SCX, SAX, High pH and SEC) and SWATH-MS were utilized to uncover protein biomarkers from a cohort of 100 plasma samples (i.e., pools of 20 healthy and 20 stages I–IV CRC plasmas). The differentially expressed proteins were analyzed using ANOVA and pairwise t-tests (p < 0.05; fold-change > 1.5), and further examined with a neural network classification method using in silico augmented 5000 patient datasets.

**Results:**

Ultradepletion combined with peptide fractionation allowed for the identification of a total of 513 plasma proteins, 8 of which had not been previously reported in human plasma (based on PeptideAtlas database). SWATH-MS analysis revealed 37 protein biomarker candidates that exhibited differential expression across CRC stages compared to healthy controls. Of those, 7 candidates (CST3, GPX3, CFD, MRC1, COMP, PON1 and ADAMDEC1) were validated using Western blotting and/or ELISA. The neural network classification narrowed down candidate biomarkers to 5 proteins (SAA2, APCS, APOA4, F2 and AMBP) that had maintained accuracy which could discern early (I/II) from late (III/IV) stage CRC.

**Conclusion:**

MS-based proteomics in combination with ultradepletion strategies have an immense potential of identifying diagnostic protein biosignature.

## Background

Global temporal patterns of colorectal cancer (CRC) incidence and mortality are alarming. In 2018, it is estimated that over 1.8 million patients were be diagnosed with CRC, resulting in over 800,000 deaths annually [1]. These statistics are expected to increase to ~ 2.2 million new cases with 1.1 million fatalities by 2030 [2]. This trend can partially be explained by the fact that early stages of the disease are especially asymptomatic with the majority of patients diagnosed when tumors have already invaded local lymph nodes (stage III) or metastasized to distant organs (stage IV), leading to survival rates lower than 13% [2, 3]. Surgical tumor resection in early stage disease can be both preventive and curative [4] with the 5-year survival rate of early stage I/II CRC patients greater than 90% [5]. There is therefore a substantial need to reliably, accurately and consistently diagnose CRC as early as possible.

There are a horde of stool-based tests and structural examinations [6, 7] that are in use clinically to aid early CRC detection. In developed countries, stool-based tests like gFOBT (guaiac chemical fecal occult blood tests), FIT (fecal immunochemical tests) and mt-sDNA (multi-target stool DNA tests) are distributed to most-at-risk populations (e.g., those aged 50–74 years) [8]. The gFOBT (sensitivity 62–79%; specificity 87–96%) and FIT (sensitivity 73–92%; specificity 91–97%) [6] tests rely on the chemical or immunological detection of fecal hemoglobin (Hb) respectively [8]. The mt-sDNA test, which has a lower (~ 90%) specificity, [6] identifies multiple molecular biomarkers, such as hypermethylated BMP3/NDRG4, point mutations in KRAS and the beta-actin gene as well as Hb protein [9]. However, despite extensive public health education programs worldwide, patient participation/compliance with fecal-based screening tests has rarely (if ever) exceeded 44% [6, 10, 11].

Positive fecal gFOBT/FIT test results (i.e., true or false positives) are referred to more invasive structural tests for confirmation. These structural tests include computed topographic colonography (CTC) and flexible sigmoidoscopy (FS) [6]. The efficacy of CTC and FS is restricted by exposure to low-dose radiation and incomplete examination of the proximal colon, respectively [6]. As per standard practice of care, all positive non-colonoscopic screening procedures are followed up with a confirmatory colonoscopy.

However, colonoscopy is expensive, invasive, requires unpleasant preparation and causes occasional adverse sedation morbidities as well as unavoidable infrequent mortality from adverse consequences like bowel perforation and sepsis [6]. Low compliance and sensitivity of fecal tests has compelled the investigation of potential blood tests that have a much higher compliance rate (as high as 97% in controlled studies).

Two primary classes of blood-based markers have been developed, namely DNA-based and protein-based. Tests that detect tumour-specific genetic and epigenetically-altered circulating tumour DNA (ctDNA) released from tumour cells are colloquially termed ‘liquid biopsy’ tests [12]. However, there remain some technology barriers to early clinical stage cancer screening using liquid biopsy tests. These include; secretion of negligible levels of ctDNA from small adenomas or early stage tumors meaning large amounts of blood are required, mutational heterogeneity among individual patients [13] and poor association of emerging mutational biomarkers with cancer stages and types, each of which limits use for screening early clinical stage CRC patients [14].

Of protein markers, carcinoembryonic antigen (CEA) was one of the earliest to be used clinically, although it has been subsequently discounted as efficacious for early-stage screening [15]. Plasma CEA levels are primarily used to monitor colorectal carcinoma treatment and to identify recurrence after surgical resection, despite having a low 35% sensitivity and 87% specificity [16]. Furthermore, CEA is expressed in many other cancers [17, 18] and is not specific to CRC. Multiple other protein markers have been proposed [19], however only a few have shown translational promise. Protein-based blood biomarkers offer significant advantages that make them amenable for the development of an ideal population blood-based CRC screening test. They purport to be accurate, specific, sensitive and inexpensive [11]. Furthermore, protein-based tests offer significant advantages in translatability with current technologies and clinical laboratory practices [20]. The key, however, remains, to find a molecular protein-based biomarker (or panel) that provides better specificity and sensitivity than gFOBT and FIT, as a pre-colonoscopy screening test.

Blood plasma is a complex body fluid owing to the high dynamic concentration range of proteins found within it. The concentration range of human blood plasma proteins extends 12–13 orders of magnitude [21], with > 90% of all plasma protein content covered by a few (10 to 14) highly abundant proteins found above the mg/ml mark. These are primarily haemostatic (e.g., albumin), acute phase response proteins (e.g., serpins), lipid/protein transporters and immunoglobulins [21, 22]. The remaining low and medium abundance proteins are found at concentrations ranging from ng/ml down to pg/ml and are often derived from proteins that have leaked or been shed from tissues (including diseased cells/tissues) or that represent interleukins, cytokines or growth factors [21, 23]. These low abundance proteins potentially hold critical information regarding the health and disease status of any individual [24]. However, low abundant proteins are masked by more abundant proteins and are difficult to detect in a proteomics discovery experiments. Indeed, the repertoire of often identified disease biomarker candidates from mass spectrometry are usually categorised as general inflammatory response proteins, lipid transporters or coagulation cascade proteins [25–27]. In other words, many proteomic biomarker studies unearth proteins of unremarkable biological context, meaning that they code for disease with particularly low specificity [28].

**Fig. 1:**
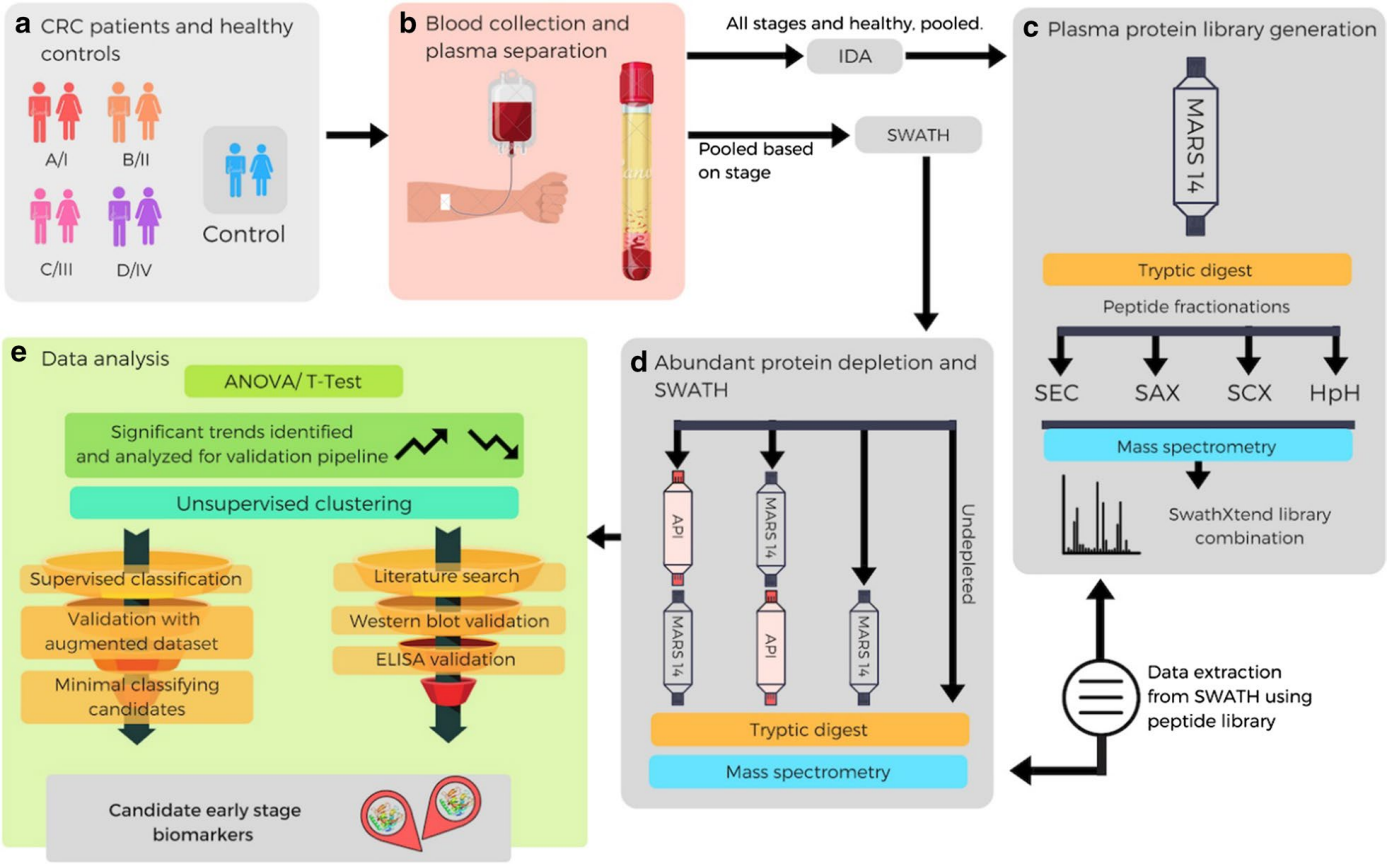
Blood-based multi-analyte proteomic signature discovery workflow: **a** A total of 100 age- and sex-matched EDTA-plasma samples were procured [n = 20 per stage I, II, III, IV, and n = 20 healthy controls (non-menopausal, non-smoking and no history of any cancers)]. **b** Plasma samples were collected as per ethics requirements. To create a plasma reference library, equal volumes of all patients and healthy plasmas were pooled. For the SWATH experiments, equal volumes of 20 plasma samples were combined to produce pools of each of the 4 CRC stages (I–IV) and healthy controls. **c** For library generation, HAPs depleted using MARS-14 column (Agilent) followed by tryptic digestion and peptide fractionation by SAX, SCX, SEC and HpH (independently), followed by IDA-MS analysis. **d** The stage pooled samples were processed through four different experiments (three, where the plasma HAP were depleted and one where it was not). The resulting proteins were digested and subjected to SWATH-MS. Lists of quantifiable proteins were extracted from the SWATH dataset using the peptide library generated in **c**. **e** Differentially expressed proteins were first identified using ANOVA/t-test (p-value < 0.05, fold change cut off ± 1.5), resulting in 37 proteins exhibited with differential expression across all CRC stages compared to healthy controls. These 37 proteins were further evaluated by unsupervised clustering method to increase discriminatory power. Differentially expressed proteins were subjected to validation pipeline where they were checked to identify evidence in the literature, followed by experimental validation (ELISA/Western blotting) of a subset that seemed most promising. Concurrently, the samples also underwent a supervised classification method which identified potential candidates which were then validated with an augmented dataset (with a SD 10 times the observed variance). This resulted in a subset of 5 candidate proteins that were able to classify the different stages of the disease. *SAX* strong anion exchange, *SCX* strong cation exchange, *SEC* size exclusion chromatography, *HpH* high pH reversed phased c18, *SWATH* sequential window acquisition of all theoretical mass spectra, *IDA-MS* information-dependent acquisition mass spectrometry, *SD* standard deviation, *HAPs* high abundant proteins

This study aimed to adopt a multilayered plasma proteomic approach to discover protein biomarkers for the detection of CRC patients at earlier stages (I/II) from EDTA plasmas. To visualise and quantify novel lower abundance proteins, we used combinations of commercially available depletion (i.e., MARS-14) [29] and an in-house ultradepletion system [30, 31]. We also employed SWATH™-MS (Sequential Window Acquisition of all THeoretical Mass Spectra) for deep and reliable exploration of the plasma proteome. These studies were applied to a set of pooled EDTA-plasma samples in order to identify potential candidates for early stage I/II CRC detection. To verify the diagnostic ability of candidate biomarkers, we performed Western blotting and ELISA on pooled and individual samples where tests were available commercially (experimental procedure summarised in Fig. 1). Finally, we utilized machine-learning approaches to further test the validity of our candidates. Unsupervised clustering algorithms were used to validate how dissimilar early stage I/II CRC were from healthy subjects. We then used supervised classifiers on generated data based on the variance found in our individual samples, which was then tested on real patient data. This discovery experiment resulted in a novel blood-based multi-analyte biomarker signature panel that requires comprehensive validation to allow population-based detection of stages I and II CRC.

## Materials and methods

### Ethics statement and sample collection

This study was performed with approval from the Macquarie University Human Research Ethics Committee (MQ HREC approval #5201200702). The cohort of 100 patient EDTA-plasma samples was procured from the Victorian Cancer Biobank (VCB) in Melbourne, Australia. The experiment assembled 100 individual EDTA-plasma samples, composed of 80 from Dukes’ staging system staged CRC (n = 20 each for stages A, B, C, and D). These have been recently clinically re-classified as stage I, II, III, and IV CRCs respectively according to the AJCC system. EDTA-plasmas were also collected from 20 healthy donors (n = 20) that had been age- and sex-matched, non-menopause and non-smoking status, all with no prior history of cancer or other major disease. Cancer and healthy plasma samples were processed identically throughout the study. All plasma samples were prepared identically as described previously [15].

### Multiple affinity removal system (MARS-14) high abundance plasma protein depletion

A previous study using the MARS-14 system has shown that depletion columns afford highly repeatable and efficient plasma fractionation with few non-targeted proteins captured [29]. The Agilent MARS-14 high capacity affinity column (4.6 × 100 mm) was designed to employ anti-human plasma protein monoclonal antibodies to remove the 14 most abundant proteins (human serum albumin, IgG, antitrypsin, IgA, transferrin, haptoglobin, fibrinogen, α2-macroglobulin, α1-acid glycoprotein, IgM, apolipoprotein AI, apolipoprotein AII, complement C3 and transthyretin) from human plasma. Depletion was performed on an Agilent 1260 HPLC system where 40 µl EDTA-plasma samples were first diluted fourfold using buffer A supplied by the manufacturer followed by 0.22 µm spin filtering at 4 °C. Eluates plasmas were injected to run on the HPLC and proteins eluted following the manufacturer’s instructions.

### In-house abundant protein immuno-depletion (API)

Untargeted proteomic analyses using current LC–MS/MS on MARS-14-depleted plasma do not efficiently reveal a multitude of low abundance, disease-specific biomarkers from human plasma [32], unlike what is observed with depleted cell proteomes. The reason for this detection disparity has been suggested to be due to the particularly steep protein abundance distribution seen with plasma versus cell proteomes. To obviate this pivotal problem, we have developed and here for the first time use an adjunct in-house “ultradepletion” method that immunodepletes additional high and medium abundance human plasma proteins [30, 31].

In detail, chicken IgY polyclonal antibodies were raised against 7 dual (SCX followed by SAX including dual flow-through proteins) ion-exchange fractions of human plasma. Purified IgYs were covalently-linked as antigen affinity-purified IgYs to activated hydrazide beads (GE, Uppsala, Sweden) following the manufacturer’s instructions and packed into columns as described previously [30, 31]. This API (abundant protein immunodepletion) column was subsequently pre-equilibrated at 5 ml/min using PBS at pH 7.2. Plasma was injected into the column at 0.1 ml/min and washed using 2.5 column volumes of PBS, first at 0.05 ml/min for 3 min and then at 5 ml/min. Bound proteins were subsequently eluted from the API column using 4 column volumes of 0.1 M glycine buffer at pH2.5 and a flow rate of 5 ml/min. Neutralization using glycine 100 mM, pH 10 was performed on all bound fractions post-elution for long-term storage at − 80 °C prior to LC–MS/MS. All samples were buffer exchanged using 3 kDa Amicon filtration and total protein quantified using a Micro BCA Protein Assay kit (Thermo Scientific™). API columns were immediately re-equilibrated with 5 column volumes of binding buffer at 5 ml/min for subsequent re-use [30, 31].

### Tryptic digestion

Prior to tryptic digestion, protein concentration was measured using a BCA Protein Assay Kit following the manufacturer’s protocol (Thermo Fisher Scientific) for both depleted and non-depleted samples. The samples were reduced with 5 mM dithiothreitol (DTT) at 60 °C for 30 min and alkylation with 25 mM iodoacetamide (IAA) at room temperature for 30 min in the dark. Samples were diluted in 100 mM ammonium bicarbonate and digested with sequencing grade porcine trypsin (Promega) at a protease to substrate ratio of 1:30 at 37 °C for 16 h. Peptide mixtures were desalted and cleaned with C18 OMIX tips (Agilent) according to the manufacturer’s protocol followed by drying by vacuum centrifugation.

### Strong cation exchange (SCX) peptide fractionation

Tryptic digested peptides (100 µg) were fractionated using a poly-sulfonylethyl column A size 200 × 2.1 mm, 5 µm, 200 Å column attached to the 1260 series HPLC (Agilent, Santa Clara, CA, USA). The separation was initiated, at a constant flow rate of 0.3 ml/min, with 100% buffer A (5 mM KH_2_PO_4_, pH 2.72, 25% acetonitrile) for 25 min. This was followed by a gradual increase in buffer B (5 mM KH_2_PO_4_, pH 2.72, 350 mM KCl, 25% acetonitrile) concentration from 0 to 45% over 70 min.

### Strong anion exchange (SAX) peptide fractionation

Digested peptides (100 µg) were fractionated using a UNO™ Q1 column (Bio-Rad, CA, USA) on a 1260 series HPLC (Agilent, Santa Clara, CA, USA). Fractionation was performed at a constant flow rate of 0.5 ml/min with peptides eluted on a linear gradient of buffers A (20 mM Tris–HCl, pH 7) for 10 min then a linear increase of buffer B (20 mM Tris–HCl, pH 7, 1 M KCl) to 100% over 60 min and held for 10 min and finally replaced with buffer C (20 mM Tris–HCl, pH 7, 2 M KCl) to 100%.

### Size exclusion chromatography (SEC) peptide fractionation

Peptides (100 µg) were fractionated using Tricorn Superdex 75 10/300 GL, 10 × 300–310 mm, 13 µm column (Amersham Biosciences) on a 1260 series HPLC (Agilent, Santa Clara, CA, USA). Elution of peptides was performed using a 100 mM NaPO_4_, 250 mM NaCl, pH 7 at an isocratic flow rate of 0.5 ml/min. Peptides were collected over 80 min.

### High pH reversed phased C18 (HpH) peptide fractionation

Peptides (100 µg) were fractionated using a ZORBAX 300 Extend-C18 2.1 ×  150 mm, 3.5 µm column on a 1260 HPLC system (Agilent, Santa Clara, CA, USA). Buffer A (5 mM ammonium formate (NH_4_COOH)) and B (5 mM NH_4_COOH, 90% acetonitrile in water) were used for the fractionation at a constant flow rate of 0.3 ml/min.

### SWATH library generation (information-dependent acquisition, IDA)

All fractionated peptides obtained from multiple peptide fractionation methods (as descripted above) were used for SWATH reference library generation (i.e., protein identification). The protein identification was performed on a Sciex TripleTOF 5600 (Sciex, Framingham, MA) coupled with Eksigent Ultra nanoLC system (Eksigent Technologies, Dublin, CA). Peptides were injected onto a reverse phase peptide C18 trap (Bruker peptide Captrap) for pre-concentration and desalted at a flow rate of 10 µl/min for 5 min with 0.1% formic acid (v/v) and 2% acetonitrile (v/v). After desalting, the peptide trap was switched in-line with an in-house packed analytical column (150 µm × 10 cm, solid core Halo C18, 160 Å, 2.7 µm media (Bruker)). Peptides were eluted and separated from the column using the buffer B (99.9% acetonitrile (v/v), 0.1% formic acid (v/v)) gradient starting from 2% and increasing to 10% for 10 min then to 35% over the next 78 min at a flow rate of 500 nl/min. After peptide elution, the column was cleaned with 95% buffer B for 10 min and equilibrated with 98% buffer A (0.1% formic acid (v/v)) for 20 min before next injection. In IDA mode, a TOFMS survey scan was acquired at m/z 350–1500 with 0.25 s accumulation time, with the ten most intense precursor ions (2+ to 5+; counts > 150) in the survey scan consecutively isolated for subsequent product ion scans. Dynamic exclusion was used with a window of 20 s. Product ion spectra were accumulated for 50 ms in the mass range m/z 100–1500 with rolling collision energy.

IDA data were subjected to database searches by ProteinPilot (V4.2, SCIEX) using the Paragon algorithm [33]. *Homo sapiens* database was obtained from SwissProt (20,204 entries, 2015 version). The search parameters were as follows: sample type: identification; cys alkylation: iodoacetamide; digestion: trypsin; instrument: TripleTOF 5600; special factors: none; ID focus: biological modifications; miss-cleavages: one; precursor peptide mass tolerance: ± 50 ppm; fragment ion mass tolerance: ± 0.1 Da; peptide length: > 7 amino acids. A reverse-decoy database search strategy was used with ProteinPilot, with the calculated protein FDR < 1% and a probability cut off at 0.99.

### SWATH-MS

A Sciex TripleTOF 5600 coupled with Eksigent Ultra nanoLC system and identical LC conditions (as described above) were used for SWATH-MS experiments. Initially, the precursor m/z frequencies from generated IDA data (above) were used to determine the sizes of m/z window. SWATH variable window acquisition with a set of 60 overlapping windows (1 amu for window overlap) was constructed covering the mass range of m/a 399.5–1249.5. In SWATH mode, TOFMS survey scans were acquired (m/z 350–1500, 0.05 s) then the 60 predefined m/z ranges were sequentially subjected to MS/MS analysis. Product ion spectra were accumulated for 60 ms in the mass range m/z 350–1500 with rolling collision energy optimized for lowed m/z in m/z window +10%.

SWATH data were extracted using PeakView (v2.1) with the following parameters: top 6 most intense fragments per peptide, fragment tolerance at 75 ppm, 10 min retention time window, confidence thresholds of 99%, FDR for transitions < 1% (based on chromatographic feature after fragment extraction) and exclusion of shared/modified peptides.

### Statistical analyses

Peptide quantification was performed using peak areas from extracted ion chromatograms and proteins were quantified using cumulative mean values of the calculated peptide quantities. The extracted data was normalized using total area normalization, and log-transformed prior to statistical analysis; the data distribution was examined using density plots and boxplots. The overall sample look and consistency of the technical replicates was examined visually using hierarchical clustering and principal component analysis (PCA) plots.

Extracted quantitation contained data from pooled samples in technical triplicates, belonging to five categories: CRC stage I–IV and healthy control. Proteins differentially-expressed between the five categories were identified based on a one-way ANOVA run separately for each protein, selecting proteins based on an ANOVA p-value criterion (< 0.05) and maximum fold change (FC > 1.5). Pairwise t-tests were also carried out, using both a protein level and peptide-level approach. The statistical analysis protocol is embedded in SwathXtend as described in detail previously [34].

### Unsupervised and supervised machine-learning

The differentially-expressed protein candidates analyzed by one-way ANOVA and pairwise t-test were consolidated in a single dataset from the different depletions, and were further evaluated, first, by being plotted in 3D-space following unsupervised clustering techniques. Dissimilarity matrix were created based on the peak areas of technical replicates for each condition, and plotted in pairwise distances by using multi-dimensional scaling. The data is represented based on the first three dimensions for each CRC stage and healthy. Results from this clustering approach were verified using PCA. Both methods were done in MATLAB.

Although supervised classification approaches have been used in recent years with proteomics datasets [35, 36], the nature of most proteomics datasets, with a high number of proteins but a small population, make their validity as early predictors of a disease debatable. One way to overcome the limitations is to generate a synthetic dataset based on our real participants’ information in order to perform classification. Data augmentation is a mainstay for training classification algorithms in the field of machine-vision and medical imaging analysis [37, 38], though not widely used yet with proteomics data. Here we adapted these methods as further validation of our results. To evaluate the predictive power of the selected panel of candidate protein biomarkers, we created a synthetic population of patients (1000 per the 4 CRC stages as well as healthy controls, total = 5000) by generating a normal distribution of random number at 10 times the standard deviation (SD) for each protein concentration from our technical replicates. Data augmentation was also performed in MATLAB.

Once the dataset was generated for each group, various classification approaches (including a shallow neural network as well as k-nearest neighbor and decision tree classifiers) were applied, using MATLAB neural network toolbox and classification app. For the shallow neural network, the network was composed of 10 hidden neurons, with 70% of the data was used for training, 15% for validation and 15% for testing. Once the network was trained, it was deployed to test on the dataset comprising our real pooled patient values.

### Western blotting

Protein concentration was measured using a BCA Protein Assay Kit following the manufacturer’s protocol (Thermo Fisher Scientific). Proteins (25 μg/sample) were separated on a 4–12% SDS-PAGE gel and transferred onto nitrocellulose membrane blots using semi-dry blotting system (Bio-Rad) following the manufacturer’s protocol. To ensure the equal protein loading in each lane, the blots were stained Ponceau S (Sigma) and imaged on a ChemiDoc™ imaging system (Bio-Rad). Blots were then incubated with primary monoclonal/polyclonal antibodies including CFD (R&D systems, AF1824, 1:2500), GPX3 (R&D systems AF4199, 1:200), CST3 (Abcam ab133495, 1:13000), PON1 (Abcam, ab92466, 1:5000), MRC1 (Abcam ab195193, 1:1000) and COMP (Abcam, ab74524, 1 :200), followed by respective HRP-conjugated secondary antibodies. Blots were imaged using a Li-Cor Odyssey Blot imager (LI-COR Biosciences). Quantitation of signal intensity of the bands in Western blots was performed using Image lab software version 5.0 (Bio-Rad) and Image Studio Lite version 5.2 (LI-COR Biosciences).

### Enzyme-linked immunosorbent assay (ELISA) validation

Expression level of ADAMDEC1 from pooled and individual plasma (n = 100, 20 per stage (I–IV) and 20 healthy control) was measured using MyBioSource ELISA kit (Catalogue #: MBS928931) following the manufacturer’s instructions. Optical densities were measured at 450 nm and 570 nm using a PHERAstar^®^ microplate reader (BMG Labtech). Statistical significance of differential expression of the plasma proteins was analyzed by one-way ANOVA on Prism software v.7 (graph pad).

## Results

### Plasma SWATH library generated using several protein/peptide fractionation methods

Protein quantification by SWATH-MS typically relies on the quality of previously-generated spectral reference libraries (i.e., SWATH libraries) for reliable peptide identification subsequent protein expression level inference. Consequently, the quality and coverage of these reference libraries are directly correlated with the efficacy and scope of finding potential candidates from any SWATH-MS analyses [39]. Particularly with complex samples like human plasma, where there are large orders in magnitude covering protein abundance, being able to obtain a large library directly influences one’s ability to quantify a greater number of proteins [21, 22].

In order to maximize the depth of SWATH reference library coverage, we strategically planned experiments as following. Firstly, we combined healthy/CRC plasma samples (n = 100) to cover all proteins present under both healthy and disease conditions. Secondly, we removed the 14 high abundant proteins (HAPs) using Agilent’s MARS-14 columns from combined plasma samples to reduce the orders of magnitude of protein concentration. Finally, after tryptic digestion of MARS-14-depleted plasmas, we employed a series of different peptide fractionation methods, incorporating reversed-phased hydrophobic interaction (e.g., HpH), size exclusion (e.g., SEC) and cation/anion exchanges (e.g., SCX and SAX). This wide range of chromatographic peptide fractionation strategies ensures maximum possible peptide coverage and hence deepest protein identification.

**Fig. 2:**
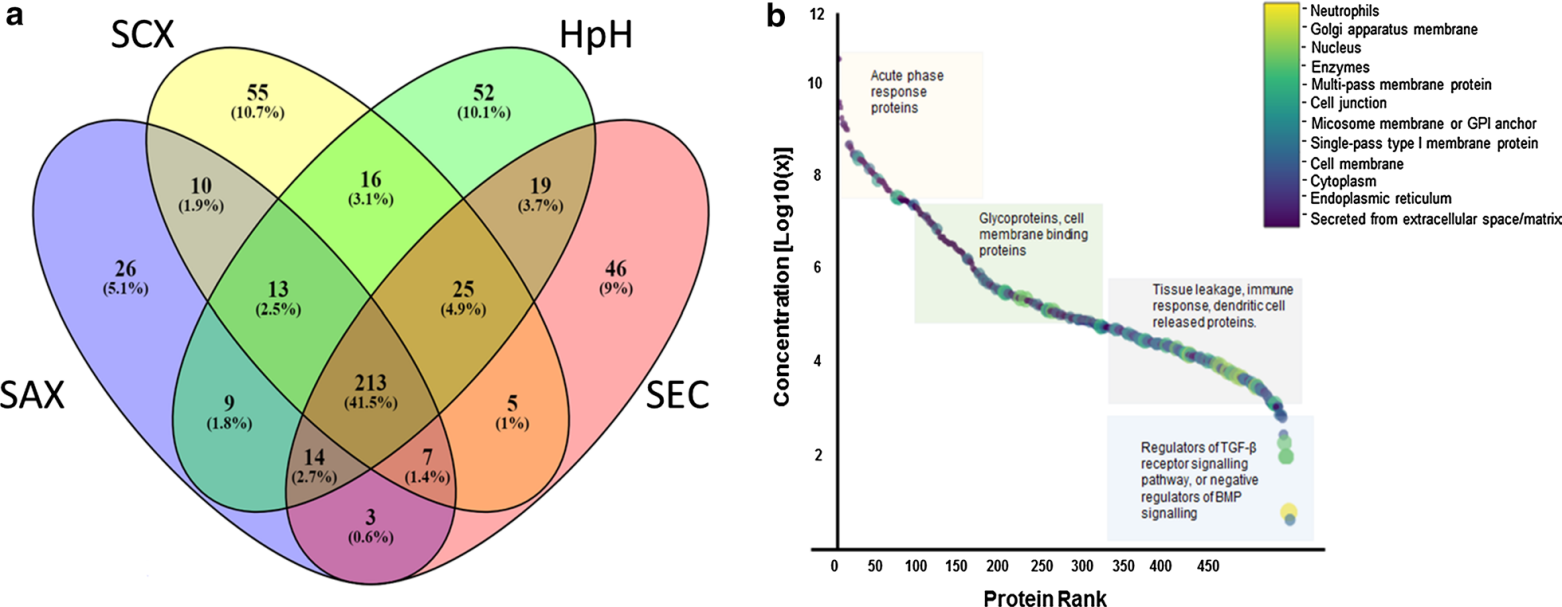
SWATH reference library with functional annotations; **a** Venn diagram [43] comparing a number of common, unshared and shared proteins identified between four peptide fractionation methods used to compile a plasma SWATH library, with **b** “Anderson curve” superimposed with gene ontology information from plasma proteins identified in the study. The color code bar shown indicated on the right-hand side of **b** corresponds to various gene ontology characteristics applied to data points shown on the concentration curve. *HpH* high pH C18 reversed phase separation, *SAX* strong anion exchange, *SEC* size exclusion chromatography, *SCX* strong cation exchange

We identified a total of 513 distinct plasma proteins by combined healthy/CRC plasma using HAP depletion and four peptide fractionation methodologies (Fig. 2a). We identified 361 plasma proteins using HpH fractionation, 295 proteins by SAX, 332 proteins by SEC and 344 by SCX. The HpH peptide fractionation method identified a most number of proteins with higher stringency MS-based identification criteria [40] (Additional file 1: Fig. S1). Detailed information for peptide/protein identification is shown in Additional file 2: Table S1 which include (i) list of proteins/peptides identified in each fractionation method, (ii) amino acid sequences of each peptides, (iii) peptide modification and missed cleavages information and (iv) neXtProt based uniqueness (uniquely mapping non-nested) of each peptide (Additional file 1: Fig. S1 and Additional file 2: Table S1).

To visualize the detectable threshold of plasma proteins in our SWATH library, we plotted a scatter plot analogous to the “Anderson curve” [21] that exemplifies the high dynamic plasma protein concentration range (Fig. 2b). Based upon the Plasma Proteome Database [41], PeptideAtlas and the PubMed literature, we were able to find reported concentrations for 427 proteins (out of 513 total identified proteins). These reported concentrations were used to create a scatter plot (Fig. 2b). It should be noted that we did not plot all 3509 human plasma proteins identified to date at high stringency by the Human (Plasma) Proteome Project [42]. It should also be noted that the 427 proteins we uncovered spanned ~ 10 orders of magnitude in protein concentration. The concentration for the most abundant protein (human serum albumin; ALB) was found to be ~ 40.6 mg/ml down to the lowest protein identified at 4.3 pg/ml which was found to be multiple EGF-like domains 8 protein (MEGF8), a protein whose function is unclear but may be involved in cell adhesion/attachment (Fig. 2b, Additional file 3: Table S2). A significant residual 86/513 human plasma proteins identified in the SWATH library currently have no reported plasma concentrations, to the best of our knowledge. Interestingly, based on search against the PeptideAtlas database on May 2019, 8 plasma proteins found in our SWATH library compilation were reported as plasma proteins for the first time (Additional file 4: Table S3).

### Functionalities of identified plasma proteins

To visualize the functionalities of proteins found in our plasma SWATH library, UniProt was employed to annotate; (i) subcellular localization, (ii) tissue specificity, (iii) gene ontology analyses (biological processes, cellular component, molecular function), and (iv) protein families (Additional file 3: Table S2, Fig. 2b). As expected, those proteins found to lie in the high abundance range were mostly classical plasma proteins such as those that are known to be liver-derived or acute phase response proteins, including HAPs like human serum albumin, immunoglobulin (multiple types), fibrinogen, chylomicron proteins, transferrin, haptoglobin, C-reactive protein, clusterin (ApoJ), and complementary factor B. Gene ontology analysis classifies these proteins as involved in biological processes like positive/negative activators of acute phase response, antimicrobial response, blood coagulation or complement activation.

Mid-range proteins, on the other hand, consisted predominantly of peptidases, serpins, S-100 family proteins, glycoproteins, and cell membrane binding proteins like cystatin C, CD59, C1Q, extracellular matrix proteins and superoxide dismutase, amongst others. Some of these plasma proteins were found to have roles in cell–cell signalling, angiogenesis and activation of MAPK activity.

In the low abundance range, cell membrane proteins, extracellular exosomal proteins, proteins secreted from the endoplasmic reticulum or lysosome membrane and intracellular secreted proteins were found. Examples included, hyaluronan-binding protein 2, galectin-3-binding protein, phosphatidylinositol-glycan-specific phospholipase D. The lowest discovered plasma proteins found were in the ρg/ml concentration range and included the E3 ubiquitin-protein ligase TRIM33 that is known to be specifically expressed in colon adenomas and adenocarcinomas and is thought to be a regulator of TGF-β receptor signaling pathway [44]. A detailed list of the SWATH library specific peptides, their length, number of peptides per proteins and their uniqueness (uniquely mapping non-nested) can be found in Additional file 2: Tables S1 and Additional file 3: Table S2.

### Identification of quantifiable plasma proteins in healthy or CRC plasmas using various (ultra)depletion strategies

Identifying specific and sensitive diagnostic biomarkers by proteomics analysis of human plasma has always been challenging [21, 22], primarily due to current LC–MS/MS methodologies not allowing detection of lower abundance disease-associated biomarkers [29] as discussed earlier. To broaden the scope of plasma protein quantification with a view to finding novel early stage CRC-specific protein biomarkers, we undertook analysis of data from a combination of strategies including non-depletion, HAP depletion and ultradepletion of both high and medium abundance proteins.

**Fig. 3:**
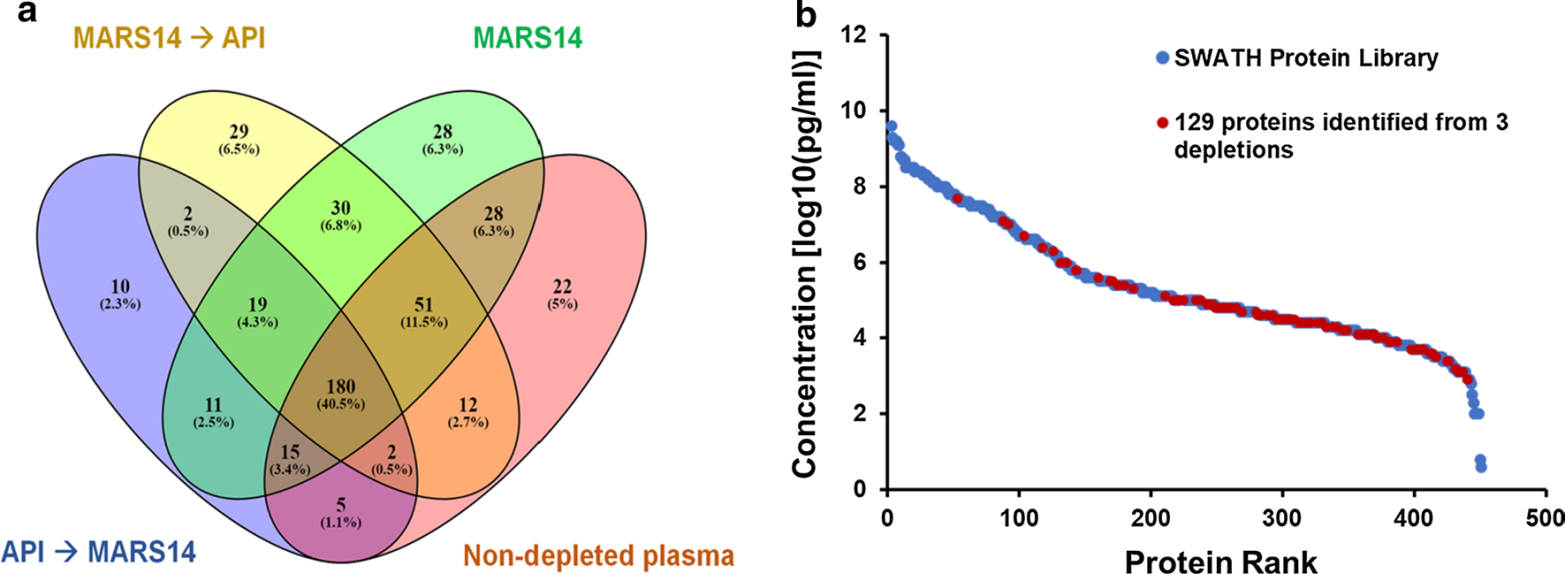
Quantifiable plasma proteins found in healthy/CRC plasmas from non-depleted and multiple plasma protein depletion strategies. Venn diagram [43] **a** showing the numbers of unique and common quantifiable proteins following three depletion (MARS-14, API followed by MARS-14 and MARS-14 followed by API) and non-depletion experiments. Protein concentration range (**b**) of the additional 129 proteins found after high-medium abundance protein depletion on the plasma SWATH library “Anderson curve”

Having compiled a comprehensive SWATH reference library, we performed the SWATH-MS analysis on pooled human healthy and CRC plasma samples. As described, pooled (n = 20) human plasmas for each of stages I–IV CRCs and healthy controls were (i) non-depleted, (ii) MARS-14 only depleted, (iii) ultradepleted using MARS-14 followed by API using purified anti-human plasma fraction chicken IgY columns [30, 31] (MARS-14 → API), and finally (iv) ultradepleted using API-depletion followed by MARS-14 (API → MARS-14). Each of the non-depleted, depleted and both ultradepleted experiments were run as technical triplicates (refer to Fig. 1 for an overview of the experimental plan). Compilation of all SWATH-MS experiments as outlined above, resulted in the identification and quantitation of a total of 444 distinct human plasma proteins from healthy or CRC plasmas (Fig. 3a). Detailed information of all quantifiable plasma proteins captured by non-depletion and depletion strategies are illustrated in Additional file 5: Table S4.

When non-depleted plasmas were analyzed, we identified and quantified a total of 315 proteins that had been deposited prior into the SWATH library. In agreement with previously published studies [29], use of the Agilent MARS-14 system that removes 14 most highly abundant plasma proteins allowed for the identification of 362 proteins, including an additional 86 plasma proteins not observed in non-depleted plasmas. Equally, non-depleted plasmas contained 41 unique proteins not found after MARS-14 depletion, indicating the distinct possibility of significant co-depletion as an off-target effect of the use of MARS-14 depletion. This observation correlates with previous work illustrating additional proteins are likely bound to MARS-14 proteins and are unexpectedly/inadvertently co-depleted [45].

To comprehensively expose lower abundance proteins differential-expression between healthy and clinically-staged CRC plasmas, we undertook various ultradepletion approaches. Systematic depletion of high-medium abundance proteins performed using MARS-14 followed by API identified 325 proteins. Of these 31 proteins had not been previously observed in non-depleted or MARS-14 depleted plasmas with 29 were not seen by any other method. When we reversed the order of ultradepletion (i.e., API depletion followed by MARS-14) we identified only 244 proteins, 12 which had not been previously observed in non-depleted or MARS-14 depletion whilst only 10 were newly identified.

In summary, MARS-14 depletion allowed 28 unique proteins to be observed whilst ultradepletion allowed for the visualization of 41 unique proteins (Fig. 3a). Collectively, we were able to identify and quantitate an additional 129 proteins (i.e., ~ 30% of the total 444 plasma proteome subset identified) using all (ultra)depletion strategies employed.

To visualize the protein concentration range of these additional 129 proteins, we superimposed them (red dots) onto the complete plasma SWATH library (blue dots) on an “Anderson curve” (Fig. 3b). This result demonstrates that these additional 129 proteins represented mostly medium–low abundance plasma proteins (e.g., LECT2, ADAMTS13 and PCDH12). These results strongly support the hypothesis that high-medium abundance plasma protein depletion allows for even deeper and more comprehensive (though not complete) proteome coverage.

### Differentially-expressed plasma protein biomarkers of early stages I/II CRC

Discovering suitable diagnostic candidates requires stringent scrutiny of large proteomics datasets using comprehensive normalization and statistical analysis. Prior to statistical analysis, the extracted SWATH dataset from each depletion and the non-depletion experiment was independently normalized using total area normalization and data distribution was examined using density plots and boxplots (Additional file 1: Fig. S2). Furthermore, consistency of sample replication was examined visually using hierarchical clustering and PCA plots (Additional file 1: Fig. S2).

To discover plasma proteins that were differentially-expressed between healthy and staged I–IV CRC plasmas, one-way ANOVA and Pairwise t-test at both the protein and peptide levels were employed. All differentially-expressed proteins were selected based-on a *p*-value < 0.05 and a fold change ratio cut off of ± 1.5. These proteins were further filtered to retain only those candidates that exhibited consistent trends (up or downregulation) in all stages compared to control, and these results were consolidated from all depletions. This analysis resulted in the identification of a total of 37 protein candidates that exhibited differential (↓↑) expression in all the four (I–IV) CRC stages when compared to healthy controls from a comparison of the non-depleted and three depleted experiments. Detailed information about each of these 37 CRC biomarker protein candidates is presented in Additional file 6: Table S5.

**Fig. 4:**
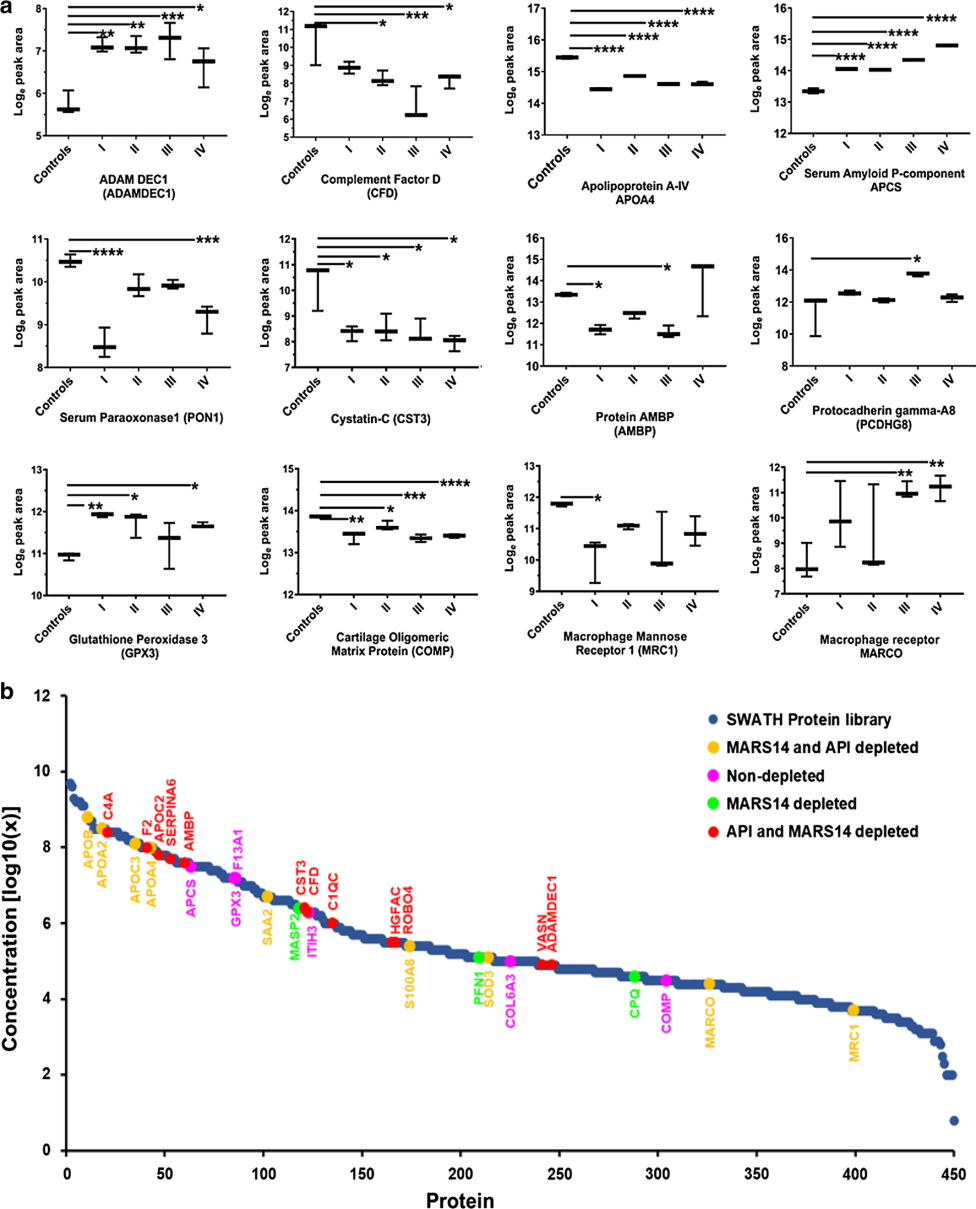
Graphical representation of differentially-expressed plasma proteins between all CRC stages (I–IV) compared to healthy controls. **a** Box plots for differentially-expressed proteins between healthy control and CRC stages I–IV. *p < 0.05, **p < 0.005, ***p < 0.0005 and ****p < 0.0001 calculated using unpaired t-tests. Distribution (**b**) of 31 potential candidates identified from four biomarker discovery experiments superimposed on the SWATH reference library protein concentration curve plotted against protein abundance rank. The color key on the top-right side shows proteins identified from different biomarker discovery experiments

The highest number of differentially-expressed proteins were found in the API → MARS-14 ultradepleted healthy against CRC samples, whereas non-depleted samples resulted in the lowest number of differentially-expressed proteins. It should be noted that some proteins (e.g., SAA2) were consistently up-regulated in disease CRC plasmas whether the data came from non-depleted or after MARS-14 depletion. Equally, GPX3 was consistently up-regulated in both MARS-14 depleted and MARS-14 → API depletion experiments. Additionally, CST3 and CFD were consistently down-regulated in all stages of CRC plasmas using both MARS-14 and API → MARS-14 depletion. Figure 4a represents a subset of these data. CRC biomarker candidate proteins were subsequently selected based on biological relevance as well as statistical analysis (e.g., predictive modelling) discussed below.

Of the 37 CRC protein biomarker candidates, 31 had reported known concentration whilst the plasma concentration of the remaining 6 proteins had not been reported. These 31 reported proteins were mapped onto the plasma SWATH library Anderson concentration curve (Fig. 4b), demonstrating that the concentrations of protein candidates were widely represented across a broad plasma protein concentration range.

We also used gene ontology characteristics of the 37 CRC protein biomarker candidates using UniProt and the Human Protein Atlas to determine potential biological relevance. Of these, 10 proteins were found to be liver-derived proteins (APOA2, APOC3, F2, APOC2, SERPIN6, PON1, AMBP, SAA1, SAA2, and HGFAC), and *in toto,* all 37 proteins had subcellular attributes associated with the cytosol (APOB, SAA1, HGFAC, S100A8, PFN1, APOA2, F2), exosomes (VASN, COMP), secretory proteins (COMP, ADEC1, SODE, HGFAC, C1QC, ITIH3, CFAD, MASP2, SAA1, SAA2, GPX3, SAMP, AMBP, PON1), or had been shown to be an integral component of cell membranes (VASN). Three candidates were expressed in somatic tissue (MECP2), endothelial cells (ROBO4) or were known to be secreted in response to dendritic cell activation and maturation (ADAMDEC1; Additional file 6: Table S5).

### Validation of differentially-expressed protein candidates using orthogonal technologies

**Fig. 5:**
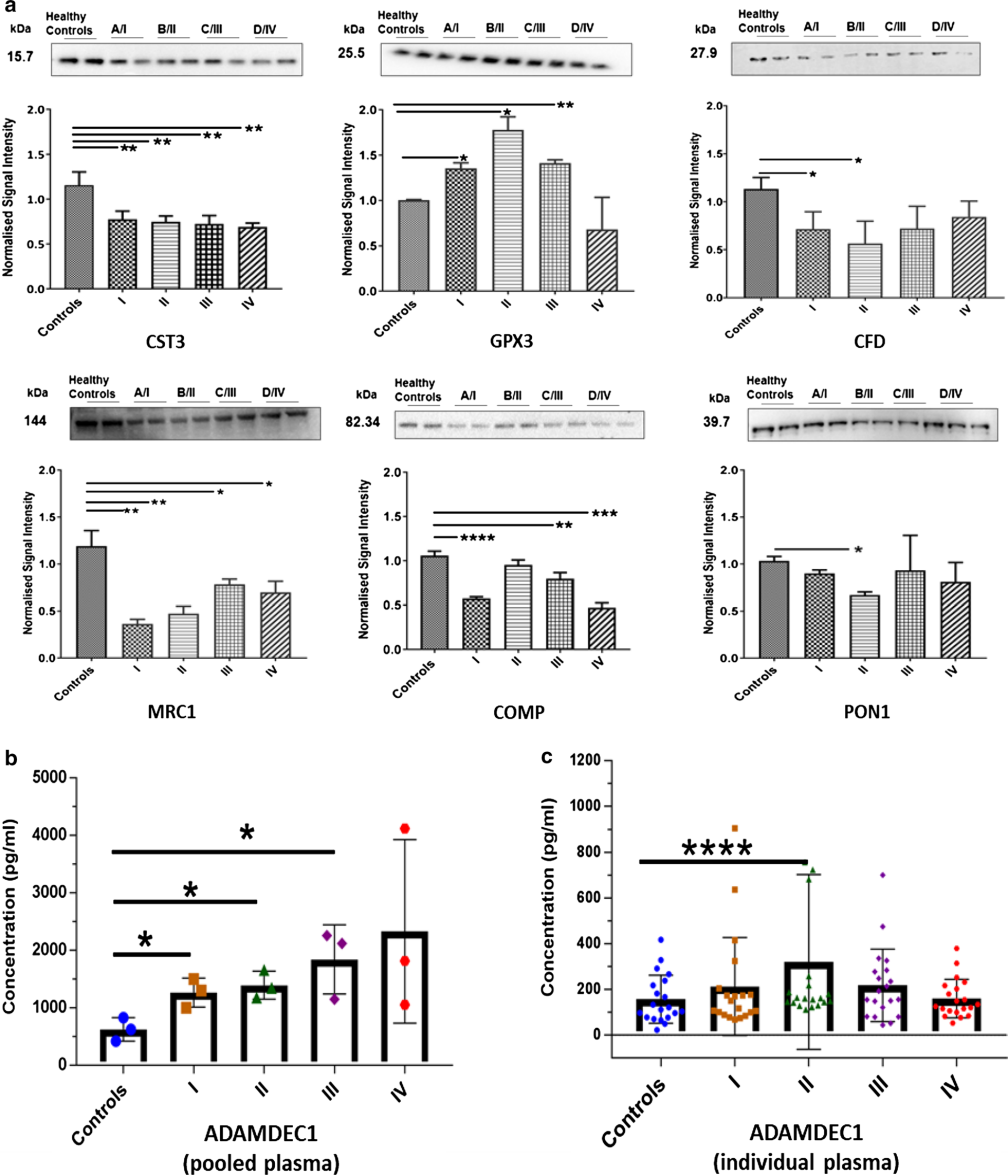
Western blotting and ELISA validation for 7 candidate early-stage CRC plasma protein biomarkers. **a** Validation of six biomarker candidates by Western blot and expression level of protein in plasma of all CRC stages (I–IV). **b** ADAMDEC1 ELISA on pooled and **c** individual patients. The bars indicate the means and SEMs. *p < 0.05, **p < 0.005, ***p < 0.0005 and ****p < 0.00005 calculated using unpaired t-test. *CST3* cystatin-C, *GPX3* glutathione peroxidase 3, *CFD* complement factor D, *MRC1* macrophage mannose receptor 1, *COMP* cartilage oligomeric matrix protein, *PON1* serum paraoxonase/arylesterase 1 and ADAMDEC1: ADAM-like decysin 1

Selected early stage CRC biomarker candidates were subsequently validated using Western blotting and ELISA. In total, 7 of 37 plasma protein candidates discovered above were validated based on previously established biological relevance in cancer, statistical analysis of data and availability of well-established, high-quality antibodies for either Western blotting or ELISA analyses. In detail, CST3, GPX3, PON1, CFD, COMP and MRC1 level variations were confirmed using Western blotting on pooled healthy and staged (AJCC I–IV) CRC plasma samples (Fig. 5a). The expression levels of ADAMDEC1 were measured using a commercially-available ELISA kit on the same pooled, as well as the individual (n = 100) healthy and staged CRC patient plasma samples (Fig. 5b, c).

Consistent with SWATH-MS results, Western blotting confirmed statistically-significant changes in expression levels of CST3, CFD, MRC1, COMP and PON1 in disease plasmas compared to healthy controls. Of these, CST3, MRC1 and COMP levels were found to be significantly down-regulated in all CRC stages in comparison to healthy, whilst the levels of CFD and PON1 were found to be significantly lower in stage I and/or stage II compared to healthy controls. Equally, GPX3 was shown to be up-regulated in AJCC stages I, II and III compared to healthy plasmas (Fig. 5a), consistent with SWATH-MS data for GPX3. Full-length Western blots and Ponceau S Acid Red stained images are shown in Additional file 1: Fig. S3. Collectively, expression levels observed in Western blotting for these 6 candidates was consistent with observed SWATH-MS quantification trends.

ELISA on pooled samples also confirmed SWATH-MS expression data for ADAMDEC1, with expression significantly elevated in stage I, II and III CRCs compared to healthy controls (Fig. 5b). However, when individual patient plasmas were analyzed by ELISA, statistically significant ADAMDEC1 expression level differences (p ≤ 0.05) were only found between stage II CRC plasmas and healthy controls (n = 20) (Fig. 5c). ELISA studies on a larger CRC population are in progress to ascertain if ADAMDEC1 SWATH differences between stage I, II and IV and healthy controls can also be substantiated.

### Neural network-based classification predicts early cancer stage using differentially-expressed CRC candidate protein biomarkers

As illustrated above, 37 differentially-expressed proteins were identified to discern early stage CRC by SWATH-MS using pooled plasma samples, rather than individual plasma samples. This approach was used to get stable population values for each stage, but also to limit the enormous cost and time requirement necessary to individually ultradeplete 100 plasma samples in an exploratory study.

An important caveat with the use of exhaustive ultradepletion and peptide fractionation methods is whether candidates identified from pooled SWATH-MS dataset (technical triplicates of pooled healthy and CRC stages I–IV) are a valid representation of the general population. Extrapolation of pooled data comports inherent risks as the variance in between participants’ plasma concentration for each candidate in unknown, neither is the variance in between candidates for a single patient. We are well aware of this limitation, but hereby propose a model to test whether our proposed candidates hold statistical power when various noise is added to our pooled data. To overcome this problem, we synthetically augmented our dataset by simulating a large number of hypothetical patients, adding noise far above (up to tenfold) the variance present in our technical replicates. This data-augmentation made it possible for us to use state-of-the-art machine-learning based statistical approaches with our dataset to test its stringency.

**Fig. 6:**
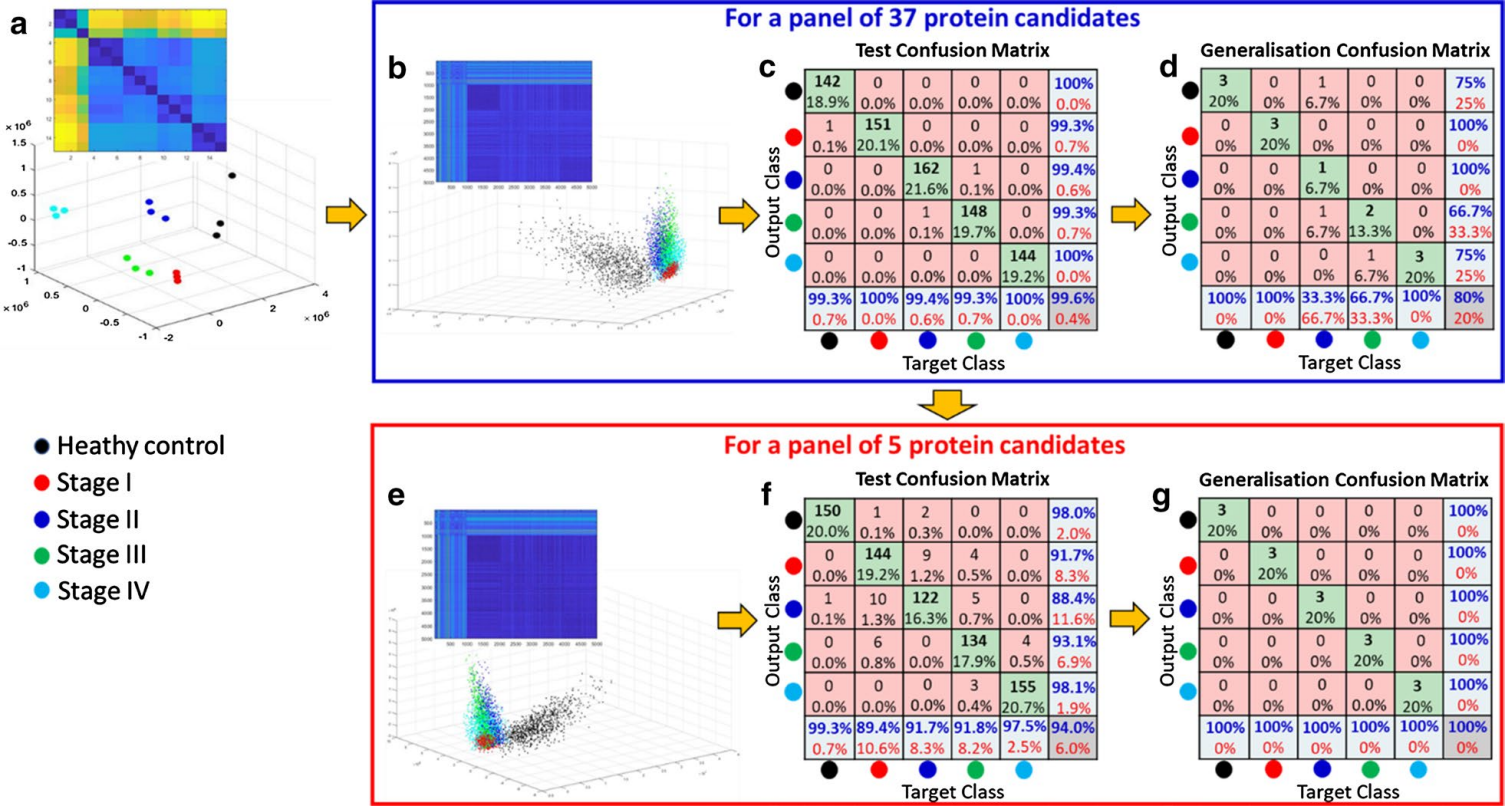
A shallow neural network-based classification of synthetic and real datasets with 37 and 5 protein candidates. **a** The dissimilarity matrix (top left corner) and multi-dimensional scaling (MDS) scatter plot for the triplicates of pooled CRC plasma samples (e.g., healthy control and stages I–IV). **b** The dissimilarity matrix and MDS plot of a synthetic dataset of a panel of 37 protein candidates. A total of 5000 synthetic patients (1000 per healthy control and the 4 CRC stages) were created from random numbers falling within a normal distribution of 10 times the standard deviation (SD) of the pooled real CRC plasma samples. **c** Confusion matrix of the synthetic dataset (for 37 protein candidates) for the test phase of the training of the classifier achieved 99.6% success. **d** Confusion matrix for the testing of the classifier on the real dataset kept out of training achieved 80% correct classification. **e** Dissimilarity matrix and MDS plot of the synthetic dataset for a panel of 5 protein candidates (SAA2, APCS, APOA4, F2 and AMB) with a total of 5000 synthetic patients. **f** Confusion matrix of the synthetic dataset (for 5 protein candidates) for the test phase of the training of the classifier achieved 94% success. **g** Confusion matrix for the testing of the classifier on the real dataset kept out of training achieved 100% correct classification

Before generating synthetic data, we verified that the variance of protein concentration from our technical replicates were similar for each stage, which they were (healthy = 33 ± 28%, stage 1 = 36 ± 34%, stage 2 = 42 ± 34%, stage 3 = 45 ± 34% and stage 4 = 31 ± 18%). We then generated a synthetic patient population of a thousand patient per (1000 patients per CRC stage and 1000 healthy subjects), and applied a conservative variance in protein expression that was 10 times that of the SD of pooled samples in absolute values over a normal distribution around the average response. Of importance, this variance was well above the observed variance of our validated individual concentrations verified by ELISA (Fig. 5c). This approach gave us the possibility to test the widest possible range of protein expression we would expect from a relatively heterogeneous population. At the same time, this approach should prevent overfitting in the training of our algorithm. As can be seen in the dissimilarity matrix per stage, our technical replicates for each CRC stage as well as for the synthetic cohort shows a clear consistency between healthy control and all 4 stages (Fig. 6a). The distinction between stages also translated well when we plotted the data using the first three dimensions following multi-dimensional scaling, with distances increasing between clusters (healthy and CRC stages) as the disease progresses from an early stage I through to more advanced stage IV.

Subsequently, we trained various supervised classification algorithms to classify each stage separately. Our trained classifier achieved 99.6% correct classification at 10 times the variance for the simulated data used (Fig. 6b, c). We then verified if the deployed algorithm could still properly classify our real dataset which was used to create the synthetic data but completely kept out of the training, and achieved 80% correct classification (Fig. 6d). This is a very encouraging validation of our candidates, and advocates progressing to population cohort studies involving measurement of each of these 37 early stage CRC candidate plasma biomarkers by targeted MRM-based approaches in individual participants to better our predictive model.

We then attempted to narrow down the number of proteins necessary to maintain high accuracy. Data-mining was performed by examining the dissimilarity distances between proteins rather than in between stages. Five proteins showed clear potential as sufficient to maintain high accuracy, which we further tested. This panel included proteins SAA2, APCS, APOA4, F2 and AMBP. Classification on our synthetic population produced a 94% correct classification from the test dataset (i.e., trained model, Fig. 6e, f) and achieved 100% correct classification once deployed on the real pooled samples that were once again kept out of the training of the algorithm (Fig. 6g). Importantly, 4 protein candidates (APCS, APOA4, F2 and AMBP) were identified from our in-house ultradepletion experiments (MARS-14 → API or API → MARS-14) whilst only 1 candidate (SAA2) was identified from non-ultradepleted experiments. This result clearly indicates the importance of plasma proteomics depth analysis for improved biomarker discovery and shows that we have very promising candidates for predicting early occurrence of the pathology.

## Discussion

Early stage diagnosis of CRC has immense actionable curative potential and has been estimated to be able to increase patient survival by > 90% [5]. Aside from poor compliance (~ 40%), stool-based testing relies on detection of blood hemoglobin in stool samples, rendering false-positive results from subjects with rectal fissures, hemorrhoids or other ailments where tissue is damaged with consequent bleeding, causing additional burden on health systems due to requisite, unnecessary follow-up colonoscopies [6]. In this scenario, blood-based testing would be undisputedly a more reliable, higher compliance (~ 97%), less invasive and more widely-accepted method of screening diagnosis. However, the discovery of reliable biomarkers with high specificity and sensitivity for early stage CRC diagnosis from blood has proven to be challenging.

### Deep dive to develop comprehensive plasma SWATH library

The most significant challenge in plasma-based biomarker discovery study is the ability to reliably and accurately measure as many as possible plasma proteins from a single experiment [21]. This is complicated by the dominance of many high abundant proteins (HAPs) that mask the identification of more biologically-relevant lower abundance proteins, which may better reflect disease pathophysiology [4]. Some antibody-based technologies (e.g., Luminex/Bio-Plex systems [46] have shown some promise, however their high cost has confined discovery to a handful of protein biomarkers. MS-based techniques have made significant recent strides with regards to accuracy and reliability and these, combined with a plethora of analytical techniques (e.g., depletion, ultradepletion, protein/peptide fractionation and IDA) can potentially tackle this challenge.

Here, we utilized a commercially available MARS-14 deletion system followed by extensive fractionation of tryptic peptides to develop a comprehensive SWATH plasma library. Although depletion of HAPs from plasma likely removed some low abundant proteins [45], it has been considered as a reliable method for discovery [47]. The plasma proteome deep dive resulted from extensive fractionation combining four [4] analytical peptide fractionation methods (i.e., HpH, SEC, SCX and SAX). All have individually been reported to be effective in peptide separation [48–50] based-on different characteristics of tryptic peptides. Collectively, our multi-fractionation approach covered a broad range of peptide characteristics. As a result, this allowed a total of 513 distinct plasma protein identifications from combined healthy/CRC plasmas (Fig. 2a) to occur. Moreover, our approach revealed 8 proteins that have not previously been identified in human plasma, searched against PeptideAtlas database (Additional file 4: Table S3). Interestingly, many of these new plasma proteins appeared to be tissue leakage proteins (e.g., CASP12, ODF3L1 and SYN2) from organs including ovary, testis and brain, respectively, most likely demonstrating these proteins are low to medium abundance in plasma. This illustrates the efficacy of the peptide fractionation method to obtain a plasma snapshot of the human body and by extension of pathophysiology.

Depletion of high abundance proteins has been previously demonstrated to allow identification of more lower abundance proteins from human plasma [47]. However, for quantification purposes, some inconsistencies have been reported [51]. To circumvent these, we utilized a strategy of using a multi-pronged approach to allow for more reliable quantitation. Here we used either MARS-14 alone, or an ultradepletion strategy with either API or MARS-14 first in tandem (Fig. 1). These approaches widened the quantifiable plasma proteome by an additional 129 proteins which were predominantly low to medium abundance proteins, demonstrated by on a plasma protein Anderson curve (Fig. 3). Our study unraveled proteins like MEGF8, CRISP3 and TRIM33 that are known to occur in lower picogram levels in plasma. Of these TRIM33 is known to be a negative regulator of BMP signaling as well as a regulator of TGF-β receptor signaling [44], whilst MEGF8 and CRISP-3 are found expressed on extracellular exosomes and are integral component of plasma membranes (Additional file 3: Table S2, Figs. 2b and 3b). These low abundance proteins sit in in lowest section of the Anderson concentration curve (Figs. 2b and 3b) belonging to G-protein coupled receptors, Notch family, interleukins, integrin beta chain family members, α and β-transferins, homeobox proteins and zinc finger proteins (Additional file 3: Table S2). Further, proteins like proprotein convertase 9, C–C motif chemokine 16, SPARC-like protein, ADAMT’s like protein 4, macrophage receptor, IgG Fc-binding protein, Golgi membrane protein 1 and ADAMDEC1 were mapped for their tissue-specific expression to colon, small intestine, epithelia and lymph nodes. These proteins are known to be involved in apoptosis, immune response, cell metabolism, cell differentiation and dendritic cell maturation respectively.

Although SWATH data does contain post translational modifications (PTMs), in biomarker discovery studies, unmodified peptides are most amenable for translation into current clinical quantitative MS-based methodologies and hence PTM differences were not studied specifically. Even though, the quantification of modified forms of proteins by SWATH is challenging, all MS data will be made publicly available for deeper investigations into the role of PTM changes in early-stage CRC and cancer progression.

### Revealed known potential CRC biomarkers

It was however not surprising to note that the subset of 37 early stage CRC differentially-expressed protein biomarkers identified through this study were observed across the entire range of concentrations represented by the Anderson curve (Fig. 4). A number of biomarker studies have previously had similar aims to this study, albeit using different samples and analytical techniques. Our study recapitulated a number of these studies that lends credence to the validity of our approach and suggest that these markers may indeed have significance.

The list of differentially-expressed proteins comprised many acute phase response proteins or those involved in the complement cascade. A number of these have been previously reported to be markers of CRC, including serum paraoxonase 1 (PON1), down-regulated in CRC plasma here as well as in other investigations [52]. PON1 is a known free radical scavenger possessing antioxidant activities and has been reported to play an important role in CRC carcinogenesis and metastasis [53]. Paradoxically, activity of sera PON1 has been demonstrated to be increased in patients with CRC [54], suggesting that a decrease in protein levels may not necessarily be associated with decreased activity, though the authors do propose that further studies are needed to be performed to validate their claims.

Plasma is the richest reserve of secretory proteins that potentially reflect abnormal physiology. Unsurprisingly, we discovered aberrations in several secretory proteins with relevance to tumor pathophysiology. The most frequently recurring marker protein was S100A8 [55, 56] found to be elevated in our study. S100A8 is predominantly expressed in myeloid cells and has been identified as a serological marker for CRC in combination with S100A9 [56]. Interestingly, Ichikawa et al., suggest S100A8/A9 promotes activation of MAPK and NF-kappa B signaling pathways and mediates tumour development [56, 57].

Another previously established up-regulated marker we unearthed was glutathione peroxidase (GPX3), an extracellular selenoprotein member known to play important roles in oxidative stress-induced apoptosis [58]. More recently, the overexpression of GPX3 has been reported in prostate cancer, gastric cancer, CRC pathogenesis and leukaemia stem cells [59–61]. Furthermore, Barett et al., had previously demonstrated that elevated plasma GPX3 may serve protective roles in inflammation-associated colon carcinogenesis by reducing oxidative DNA damage [32]. However, Roman et al., reported no significant differences between CRC and healthy control levels of serum GPX3 [62] although they were unable to validate these findings with orthogonal techniques. In our study, GPX3 was elevated across all CRC stages compared to healthy plasmas. Due to this apparent discrepancy with literature reports, we used Western blotting to validate GPX3 expression which confirmed our SWATH-MS results.

Apolipoproteins A4 (ApoA-IV) and Apolipoprotein B, both small intestine and duodenum specific proteins also stood out in the data. A recently published study established that aberrant ApoA-IV expression in CRC patients was associated with 8q24 oncogenic SNPs and with diabetes mellitus (DM) with suggestion that this protein may subsequently facilitate CRC development [63]. In our study ApoA-IV levels across all CRC stages were found to be significantly down-regulated in comparison to healthy controls consistent with past genomic studies [63]. On the other hand, elevated levels of Apo B in serum have previously been associated with CRC risk in a study performed on 28,098 participants, out of which incidence cases were identified in follow-up done from 1991 to 2012 with a 95% confidence interval [64]. This correlated with data from our study where ApoB levels were found to be significantly up-regulated across all CRC stages compared to healthy control plasmas.

A subset of biomarkers emanating from this study have been shown to be expressed in multiple cancer tissue types, including CRC. For example, cystatin C (CST3) is a secretory protein known to be a potent cathepsin B (CTSB) inhibitor [65]. It is thought that CTSB participates in remodeling of connective tissues during tumour growth, invasion and metastasis [66]. Our study found CST3 down-regulated in CRC stages, whereas a number of studies have associated up-regulation of CST3 associated with progression of cancer [67]. Several studies have suggested CST3 is not reliable, proposing alternatively that prognostic value lies in disturbances in CTSB/CST3 ratios [52, 65, 68]. Nevertheless, data here validated down-regulated levels of CST3 finding significant fold change between all CRC stages and healthy controls. However, subsequent detailed statistical modelling indicated that CST3 did not add particular value in classifying CRC tumor stage. The link between uPAR and CSTB, both being proteases is certainly intriguing and worth investigating further as both are known to be significantly up-regulated and associated with poor outcomes from CRC metastasis [69].

### Novel CRC biomarkers

Plasma proteins are largely secreted by liver and tissues through which they circulate [21, 24]. In the panel of early CRC stage candidates, it was interesting to observe changes in proteins specifically expressed in colon and associated intestinal mucosal lining tissues. Of such proteins, one interesting candidate was ADAMDEC1 which is selectively expressed and shed by maturing dendritic cells and macrophages predominantly in the small intestine, caecum and large intestine [70, 71]. ADAMDEC1, a disintegrin and metalloprotease, is a particularly unique member of ADAM family in that it lacks a transmembrane domain which allows it to remain soluble [72]. It is one of four ADAM’s released from thrombin-stimulated platelets and cleaves cell surface pro-epidermal growth factor (pro-EGF) at an arginine residue to generate soluble high-molecular weight EGF (HMW-EGF) [72]. HMW-EGF is an effective ligand for EGF receptor members and ultimately triggers the EGF signal transduction pathway [72]. A more recent study found ADAMDEC1 up-regulated in normal epithelial cells, specifically after these normal cells had been co-cultured with active mutant RasV12-transformed epithelial cells [73]. This study suggested that ADAMDEC1 may be an epithelial intrinsic soluble factor that promotes apical extrusion of RasV12 cells, displaying anti-tumour activity, in a phenomenon called “epithelial defense against cancer” [73]. In both studies, increased level of ADAMDEC1 was demonstrated to play a crucial role in tumour division and progression. However, it must be noted that, increased levels of ADAMDEC1 have also been shown to be associated with the inflammation in Crohn’s disease [71] and has also been reported to be highly expressed in Chronic rhinosinusitis with nasal polyps [74].

Here, we observed up-regulated levels of plasma ADAMDEC1 in all CRC stages compared to healthy controls and this trend was confirmed by ELISA performed on both pooled and individual patient (n = 20 per CRC stage) plasmas. This study of individual patient plasma samples allowed us to investigate the impact of “pooling” plasma samples in the first place, necessary to complete technical protocols within a reasonable timeframe. Although, pooling had advantages in discovery (discussed earlier), extrapolating protein biomarker information to individual patient populations based on that pooled data is counterintuitive. Therefore, ADAMDEC1 was used as a “example” protein to investigate the efficacy of extrapolation of pooled data for the complete list of all 37 candidates. Individual ADAMDEC1 SD values were then used to inform cutoffs for the generation of a machine learning algorithm as discussed.

Another novel finding was a subset of immune system protein biomarkers. Any human body harboring tumors likely initiates assault on physiological wellbeing. Cells of the immune system continually monitor tissues and provide protection against many types of pathology, including monitoring tumorigenesis [75]. Macrophage receptor (MARCO), a scavenger receptor is expressed by suppressive tumour-associated macrophages (TAM) called M2 macrophages. These are known to suppress the immune system favouring tumour growth and promoting metastasis through pro-angiogenesis and tissue remodelling [76]. Interestingly, Georgoudaki et al, showed targeting MARCO-expressing TAM’s enhance the effect of immune checkpoint therapy in both melanoma and CRC [75]. Macrophages are recruited to the tumor via blood circulation or direct immigration to adjacent tumors from surrounding tissues which might explain the elevated plasma levels of MARCO observed here across all CRC stages. Considerable increases in fold change ratio in later stages (C/III and D/IV) could be the result of immune suppression accelerating clinical tumour growth and metastasis. Another immunoregulatory protein, macrophage mannose receptor 1 (MRC1) also known as CD206 is an M2 marker and has been found to be co-expressed with MARCO in CRC cell lines by Georgoudaki et al, [75]. A study on advanced imaging agents found that MRC1/CD206 a C-type lectin mannose receptor is a major binding receptor for γ-tilmanocept—a compound routinely used for molecular imaging and mapping of sentinel lymph nodes [77]. In our study, MRC1 was observed to be down-regulated in all CRC stages compared to healthy controls. However, interestingly MRC1 has been reported to be up-regulated in CRC [78] although the study was only performed on small number of patients.

Just as with ADAMDEC1, it is likely that different diseases will share proteins implicated in their pathogenesis as there are > 20,000 protein coding genes and 14,500 diseases classified by the ICD code [24]. The specificity of these markers to identify the CRC as a standalone or as a panel will only be established once their diagnostic value is proven on individual plasma samples of early stage CRC patients, healthy controls and negative controls (samples from patients suffering from disease other than CRC). Panels reflecting different Hallmarks of Cancer [79] associated with a particular cancer will help ensure specificity.

We recognise that the effects of human disease on the plasma proteome are particularly complex and that it is impossible to control for all plasma changes associated with inflammatory, immunological and/or connective tissue reaction sequelae that occur as a result of confounding common disease elements.

### Predictive neural network classification reveals a subset of potential biomarkers for early CRC detection

Though ultradepletion of pooled CRC-staged plasmas allowed increased analytical depth and identification of novel low abundance proteins, it can also be a limitation if the overall end-game is to generate tangible, predictive models for high-throughput diagnosis. Machine-learning approaches are becoming more mainstream for proteomics studies [35]. These methods are often ill-suited for analysis of limited datasets from demanding, economically expensive and person-hour resource-intensive proteomics studies (e.g., where ultradepletion is performed). In a proof-of-concept experiment, we generated a synthetic patient population to train a classification algorithm and then tested this on real patient samples. We trained the algorithm assuming pooled plasma samples represented a centroid around which a normal distribution of biomarker concentrations would reside. This hypothetical variance present in human plasma protein concentration needs to be conservative, as high variability even occurs between twins over time [80]. Our supposed variance considered:

1. variance between individuals over time and environmental factors;
2. variance between technologies employed keeping in mind high-throughput testing on a population scale is our long-term aim, and
3. variance amongst clinical stages of CRC.

It is important to note that our choice of potential biomarkers was stringent and based upon orthogonal, complementary approaches with consideration of a reasonable biological rationale. With these restrictions in mind, we have managed to use as high as 10 times the SD from the mean for our generated population and maintain a near perfect classification on disease stages with our 37 candidates. High classification rates remained with as low as 5 of our proteins of interest. We therefore propose this panel of candidates as highly interesting for potential predictive purposes, and now propose to replace these generated samples with biological ones as a larger patient population dataset (individual targeted protein assays) over time. Of interest regarding the richness of selected biomarkers, progression of CRC from stage I to IV resulted in increased separation distance between stages from healthy to stage IV CRC. This fits very well with a narrative that would be expected as a condition of patients deteriorate, and biological manifestation of cancer increases.

### Next steps

PubMed searches of biomarkers for almost any disease generate hundreds of candidate results, each touting potential biomarkers of note. However due to many challenges [81] few (if any) transition to clinical practice. Most individual markers simply do not meet stringent specificity and sensitivity criteria. and recent publications encourage the use of biomarker panels [82, 83] as more efficacious than single markers. In this study, candidates from multiple depletion experiments using combinations of commercial and patented ultradepletion methods were prioritised under two layers of scrutiny. This comprised of an unbiased statistical analysis, then accounting for cancer biology and functional nature of statistically significantly and differentially-expressed proteins. We carefully prioritised and confirmed ADAMDEC1, MARCO, MRC1, S100A8, ApoAIV, GPX3, COMP, C1QC and CFD by additional study of individual patient variation, using orthogonal techniques. The potential of these proteins as a diagnostic marker panel will be further validated by measuring expression in individual healthy and staged CRC population patient samples using immunological and targeted proteomics technologies.

## Conclusions

MS-based proteomics in combination with depletion strategies have the potential of identifying multiple protein targets in human plasma. Unfortunately, the translational value of most putative markers into the clinic is abysmal. One of the ways to build that successful connection between identification, confirmation and clinical validation where the diagnostic ability of biomarker is to develop iterative methods as shown. Such methods can examine the potential of biomarkers in larger patient cohorts, and benchmark against current screening methods in an in silico fashion. We have identified a subset of 5 markers that can potentially delineate the different stages of CRC and have generated a number of hypotheses that can be tested. From a functional perspective, a couple of markers demonstrated interesting biology (MARCO and ADAMDEC1) deserve more in-depth investigation, especially validation in other cancers and non-cancer-related disease, disorders and syndromes. Evaluating these against a fresh large subset of patient data (cancer and non-cancer) would be the ideal validation strategy.

## Supplementary information

**Additional file 1: Figure S1.** Venn diagram comparison of the number of common/uncommon proteins in different peptide fractionation methods. **Figure S2.** Normalized SWATH dataset from different depletion methods. **Figure S3.** Western blotting images for selected protein candidates. **Figure S4.** Western blotting images for isotype controls.

**Additional file 2: Table S1.** List of identified proteins/peptides from different peptide fractionation methods.

**Additional file 3: Table S2.** Plasma protein concentration, subcellular location and tissue specificity.

**Additional file 4: Table S3.** List of 8 proteins that have not previously identified in human plasma, searched against PeptideAtlas database.

**Additional file 5: Table S4.** List of quantifiable plasma proteins captured by non-depletion and depletion strategies.

**Additional file 6: Table S5.** List of 37 potential CRC biomarker protein candidates.

## Data Availability

Mass spectrometry data is available through the ProteomeXchange consortium with the dataset identifier PXD014972.

## Abbreviations

AJCC: American Joint Committee on Cancer
API: abundant protein immuno-depletion
CRC: colorectal cancer
CTC: computed topographic colonography
ctDNA: circulating tumour DNA
FDR: false discovery rate
FIT: fecal immunochemical tests
FOBT: fecal occult blood test
FS: flexible sigmoidoscopy
gFOBT: guaiac chemical fecal occult blood tests
HAPs: high abundant proteins
HpH: high pH reversed phased c18
HPLC: high-performance liquid chromatography
IDA: information-dependent acquisition
MARS 14: multiple affinity removal column human 14
MRM: multiple reaction monitoring
MS: mass spectrometry
mt-sDNA: multi-target stool DNA tests
PCA: principal component analysis
SAX: strong anion exchange
SCX: strong cation exchange
SD: standard deviation
SDS-PAGE: sodium dodecyl sulfate polyacrylamide hell electrophoresis
SEC: size exclusion chromatography
SWATH: sequential window acquisition of all theoretical
PTMs: post translational modifications
