# Supplementary material for: Potential early clinical stage colorectal cancer diagnosis using a proteomics blood test panel
Source: Clin Proteomics. 2019 Aug 28;16:34. doi: 10.1186/s12014-019-9255-z (PMC6712843; doi:10.1186/s12014-019-9255-z)
Supplement: Supplementary file 1 — Additional file 1: Figure S1. Venn diagram comparison of the number of common/uncommon proteins in different peptide fractionation methods. Figure S2. Normalized SWATH dataset from different depletion methods. Figure S3. Western blotting images for selected protein candidates. Figure S4. Western blotting images for isotype controls. [file 12014_2019_9255_MOESM1_ESM.docx]

**Additional Figures**

**Potential early clinical stage colorectal cancer diagnosis using a proteomics blood test**

Seong Beom Ahn^1+^, Samridhi Sharma^1+^, Abidali Mohamedali^2+^, Sadia Mahboob^1^, William J. Redmond^1^, Dana Pascovici^3^, Jemma X. Wu^3^, Thiri Zaw^3^, Subash Adhikari^1^, Vineet Vaibhav^1^, Edouard C. Nice^4^ and Mark S. Baker^1^**^*^**

^1^ Department of Biomedical Sciences, Faculty of Medicine and Health Sciences, Macquarie University, NSW, 2109, Australia

^2^ Department of Molecular Sciences, Faculty of Science and Engineering, Macquarie University, NSW, 2109, Australia

^3^ Australian Proteome Analysis Facility (APAF), Department of Molecular Sciences, Faculty of Science and Engineering, Macquarie University, NSW, 2109, Australia

^4^ Department of Biochemistry and Molecular Biology, Faculty of Medicine, Nursing and Health Sciences, Monash University, VIC, 3800, Australia

*Corresponding Author: Mark S. Baker, Level 1, 75 Talavera Road, Macquarie University, 2109, Australia, +61 2 9850 8211, [mark.baker@mq.edu.au](mailto:mark.baker@mq.edu.au)

^+^ These authors contributed equally

Additional Information contains:

Additional Figures S1-S4


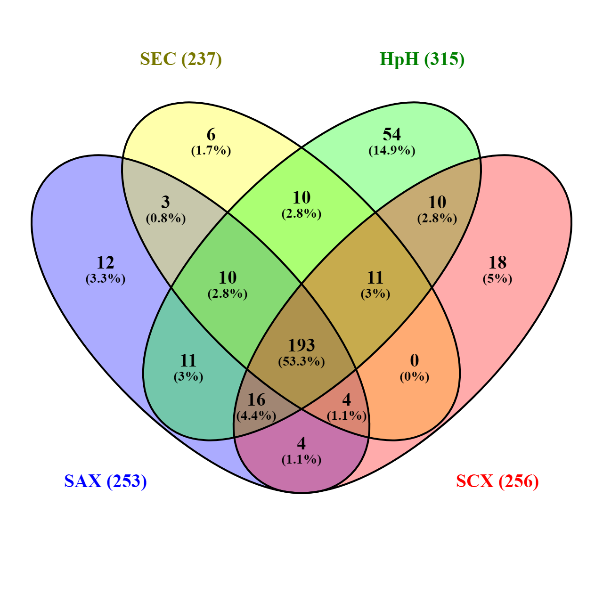


**Figure S1**: Venn diagram comparison of number of common, unshared and shared identified proteins (containing >2 uniquely mapping non-nested peptides of amino acid length >9) between four peptide fractionation methods. **HpH**: High pH C18 reversed phase, **SEC**: Size exclusion chromatography, **SAX**: Strong anion exchange, **SCX**: Strong cation exchange.


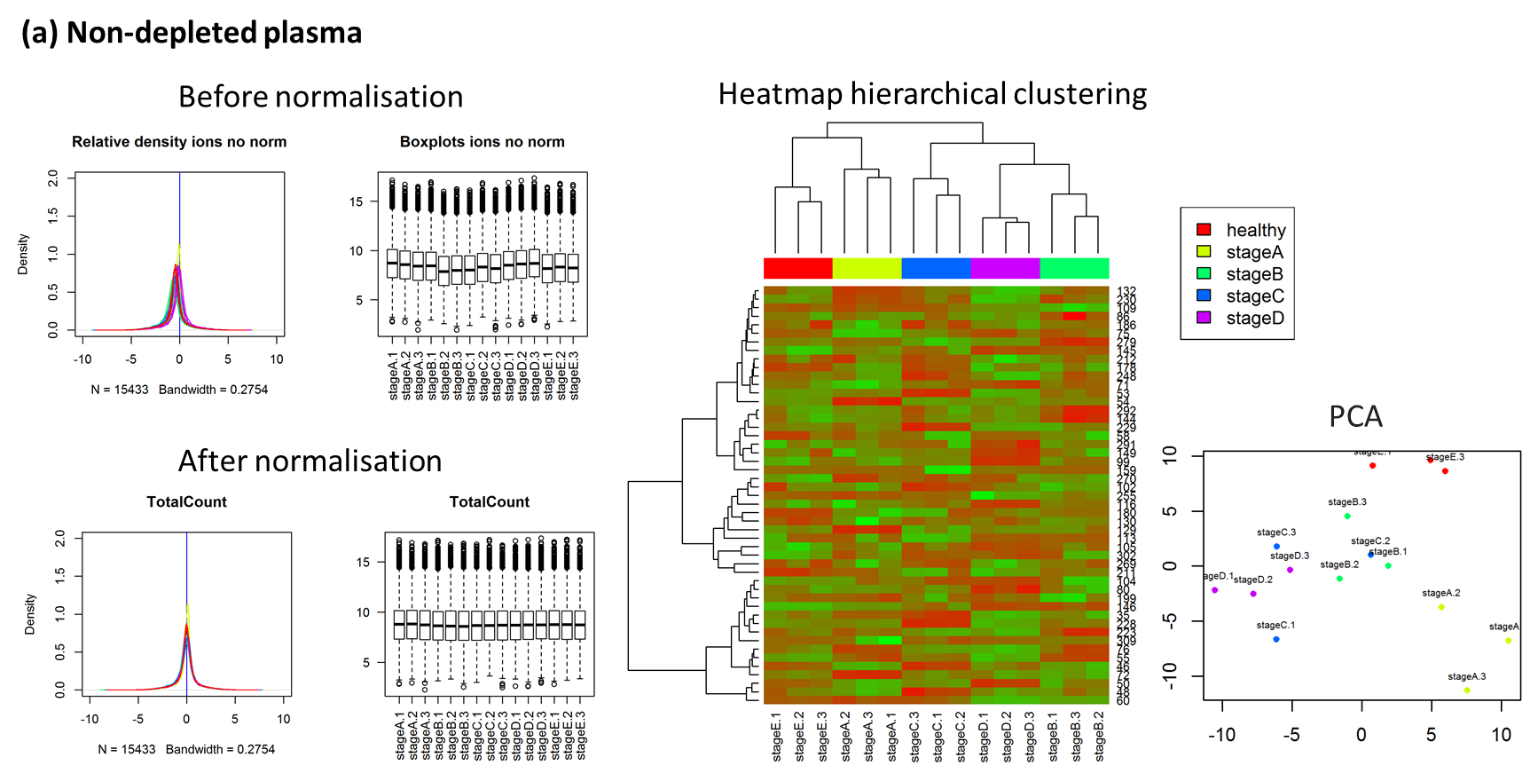

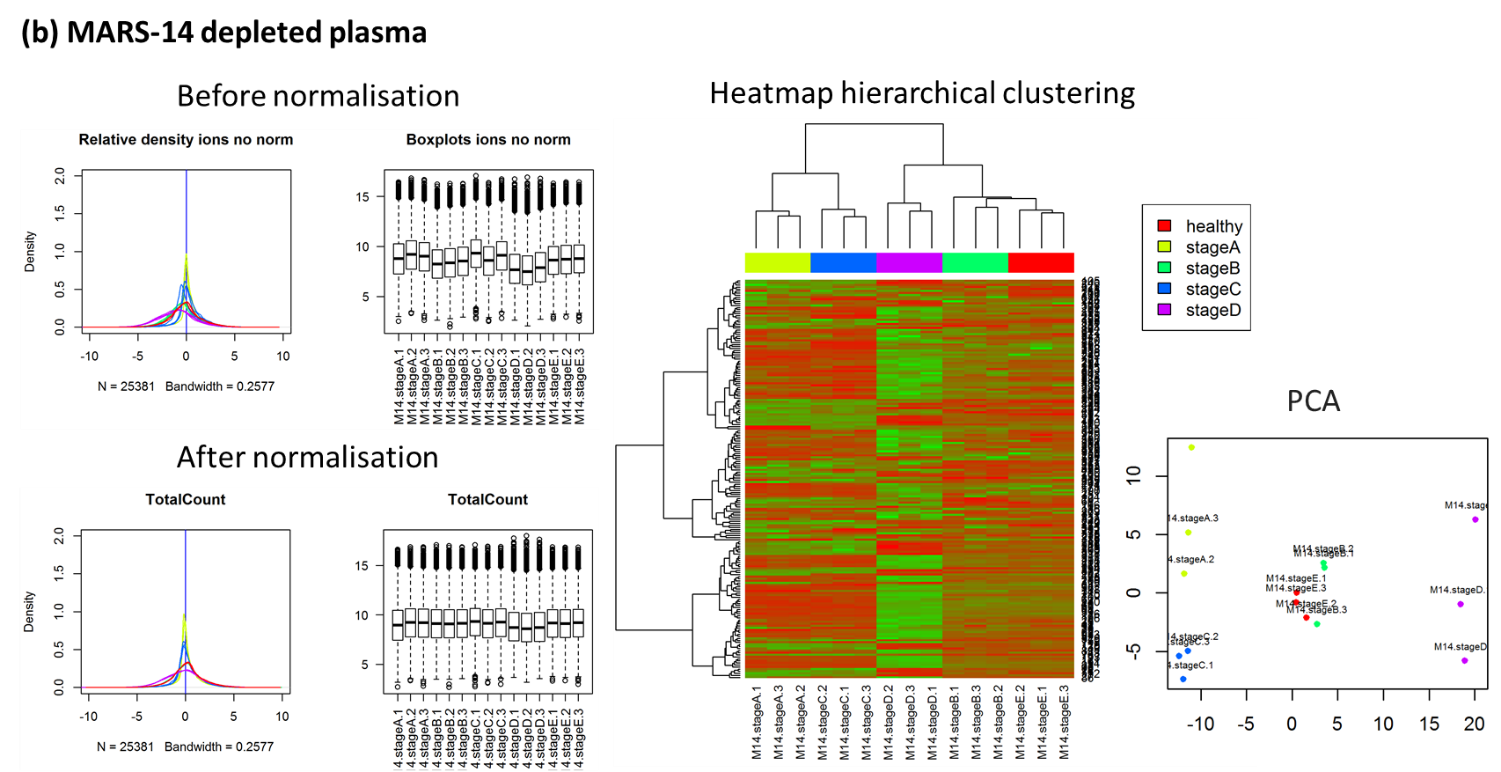


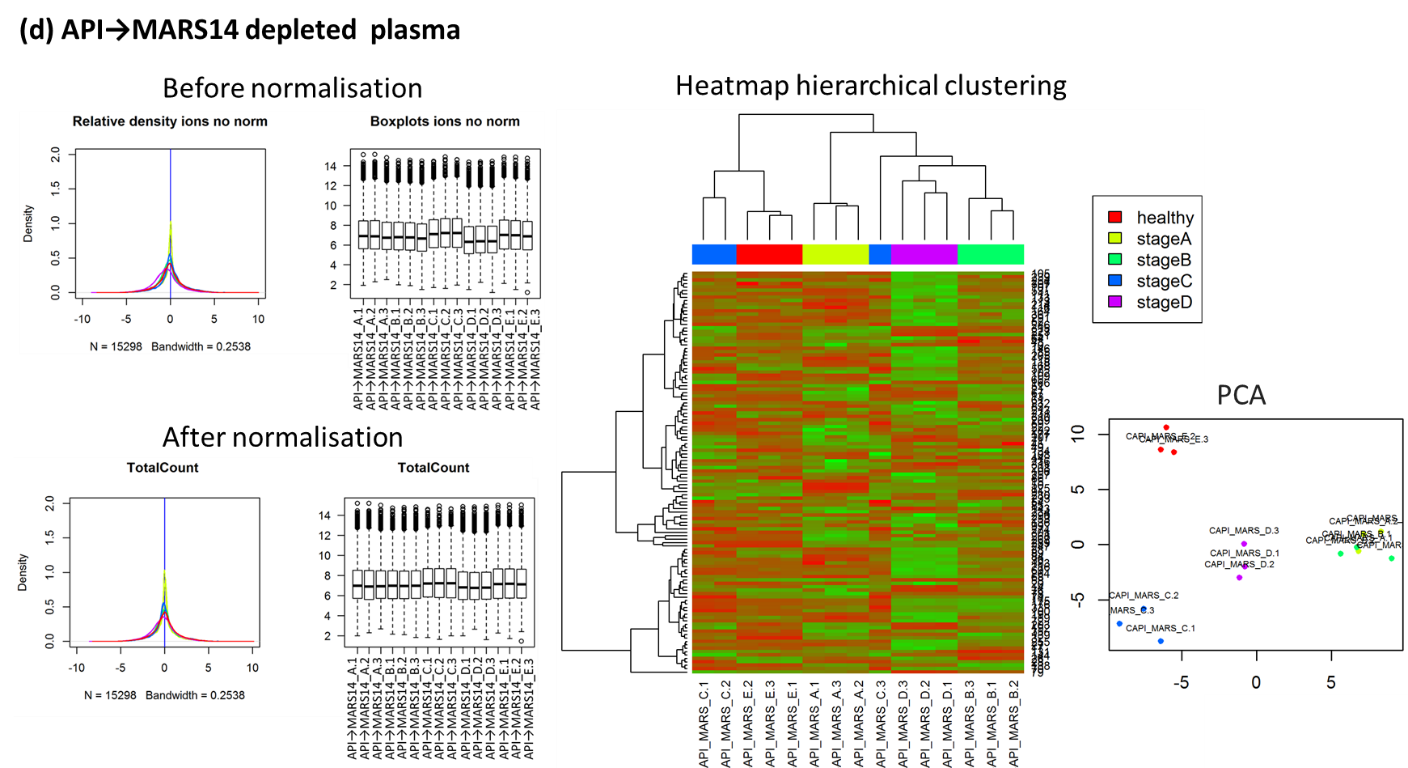

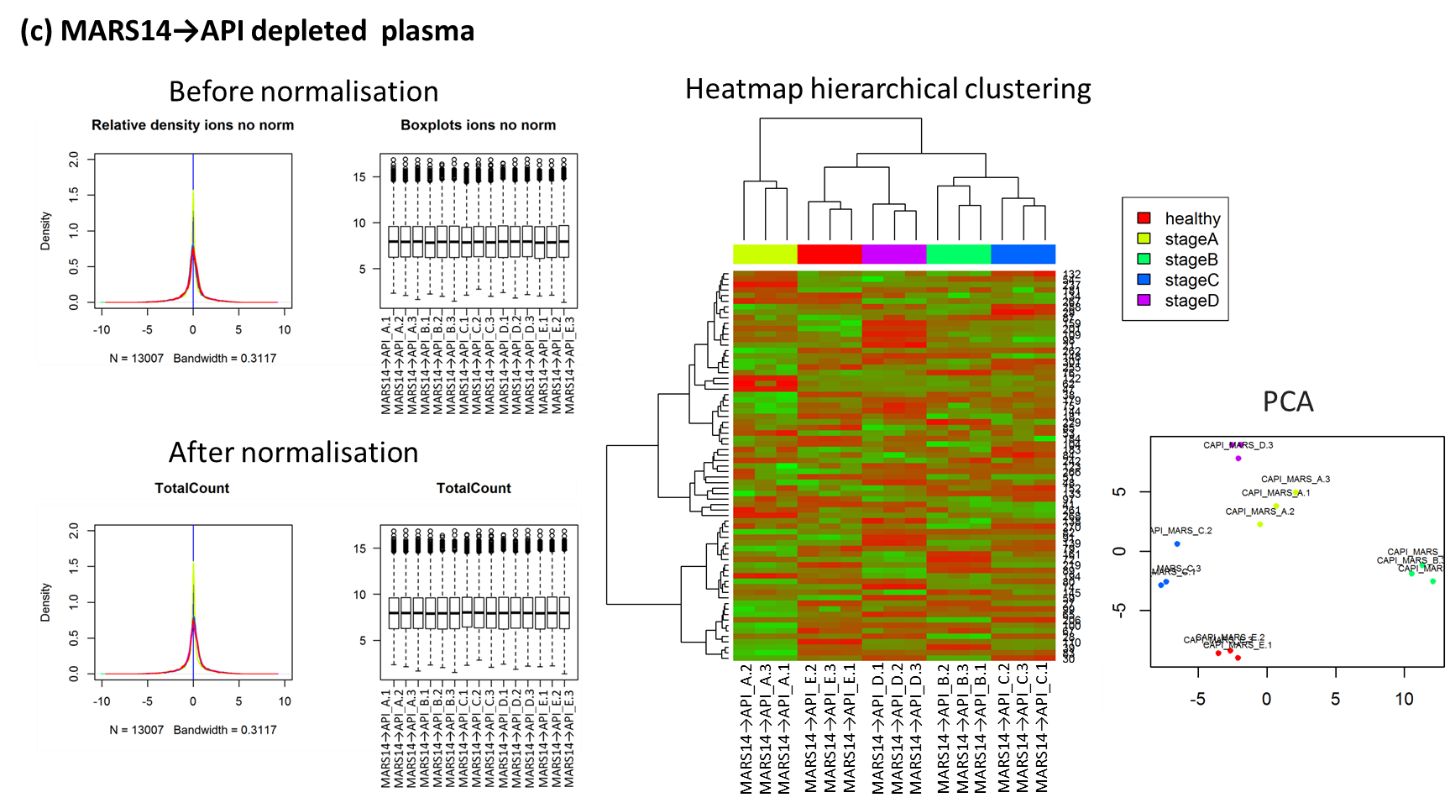


**Figure S2**: Extracted SWATH dataset from **(a)** non-depleted, **(b)** MARS-14 depleted, **(c)** MARS-14→API depleted **(d)** API→MARS-14 depleted experiments were independently normalized using total area normalization. The data distribution was examined using density plots and boxplots. The consistency of the sample replication was examined visually using heatmap hierarchical clustering and PCA plots. Healthy controls represented as stage E in the images. PCA: Principal component analysis.


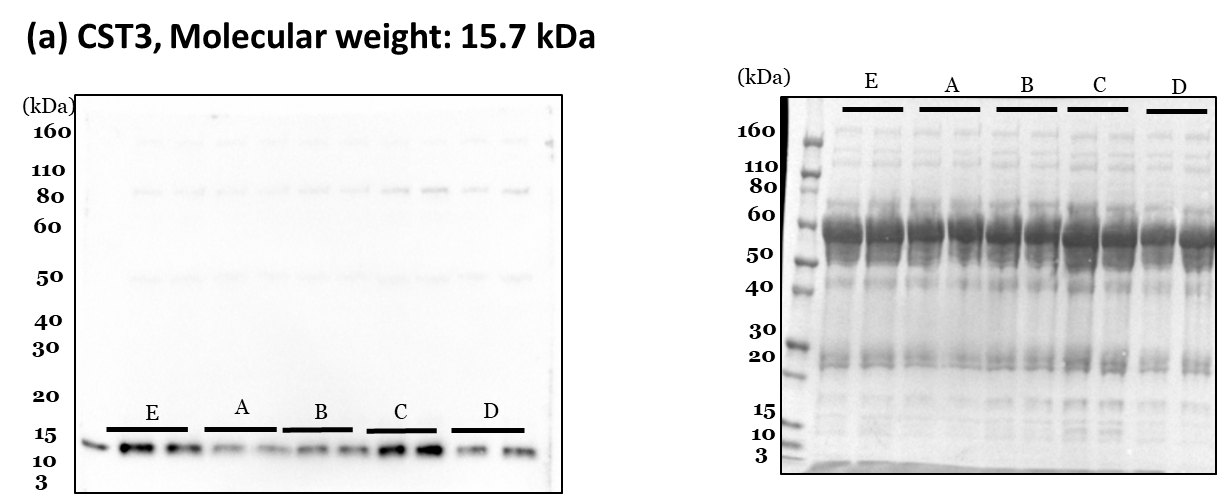


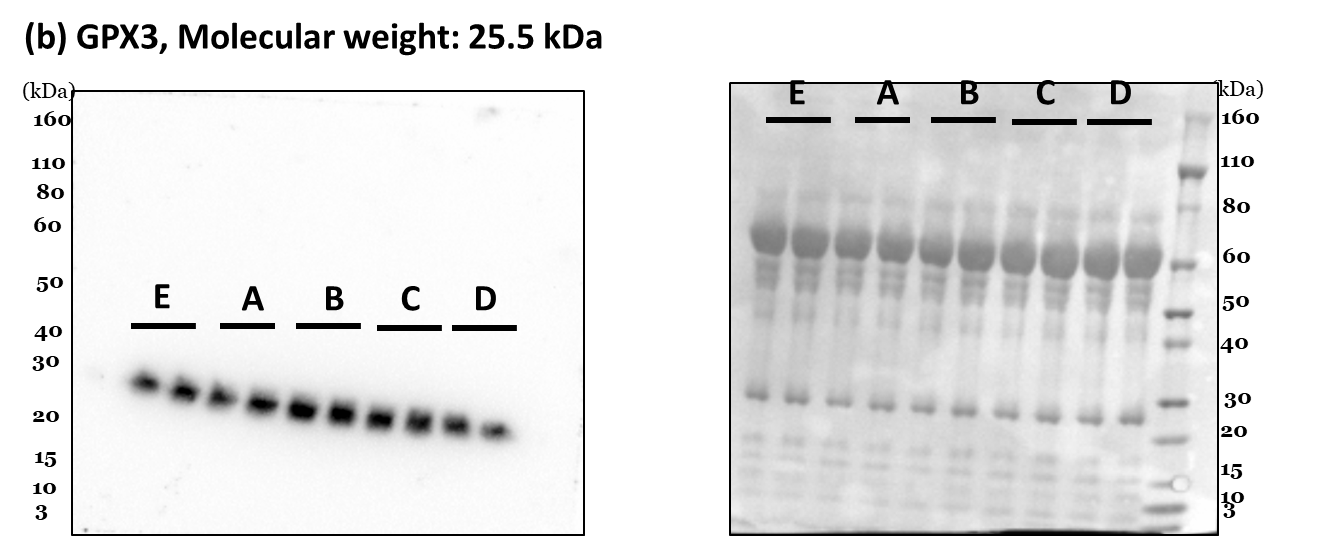


**
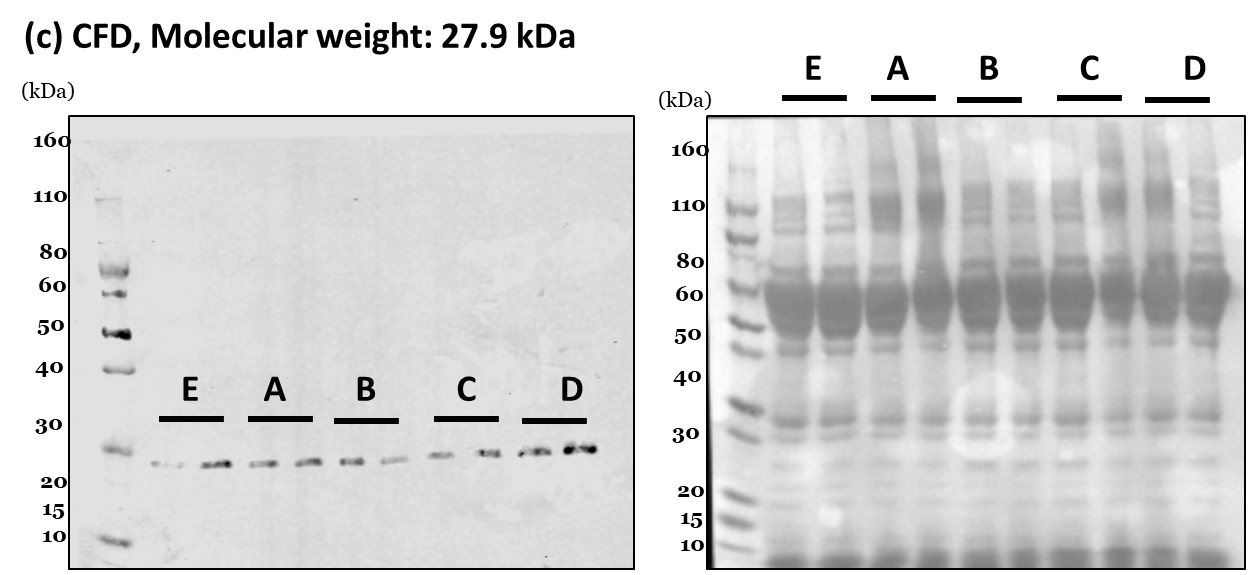
**

**
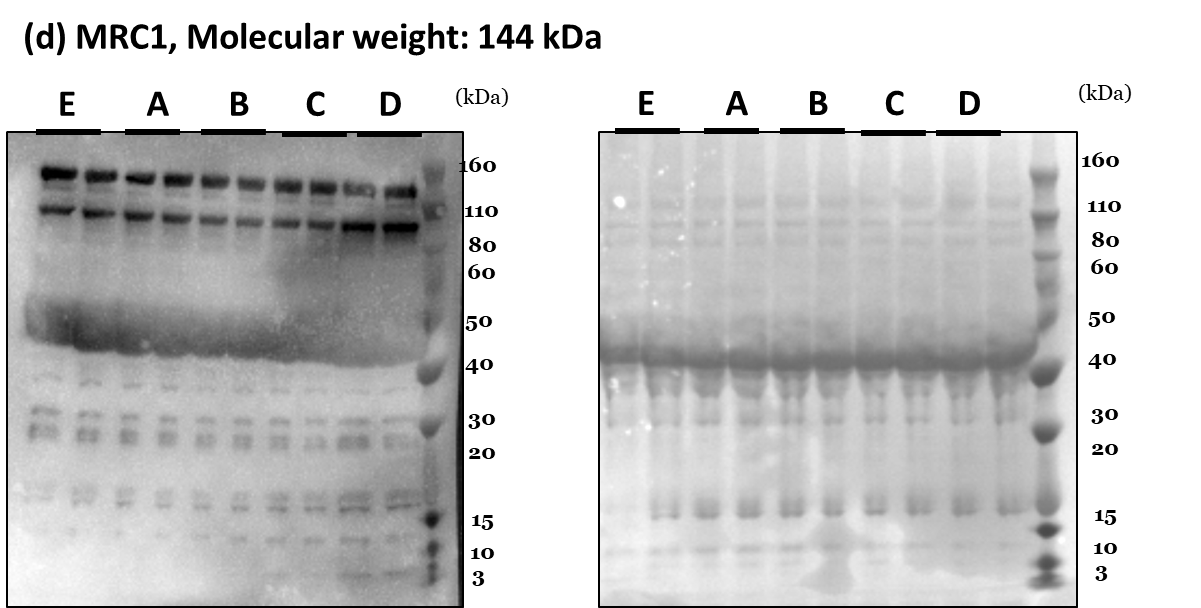
**

**
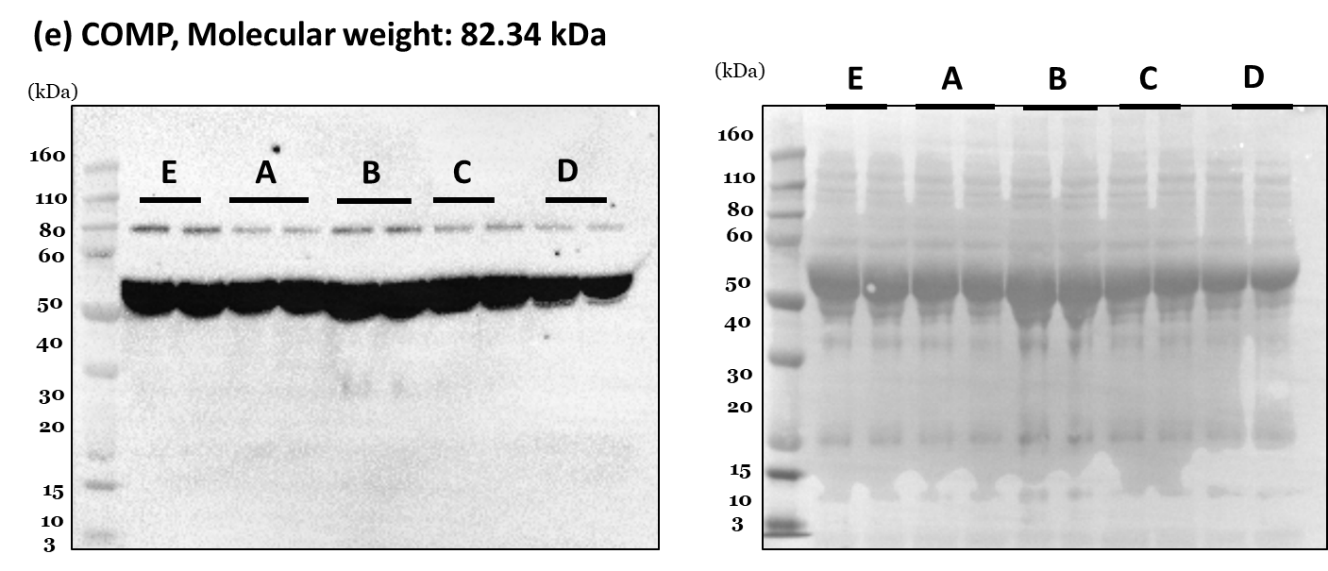
**

**
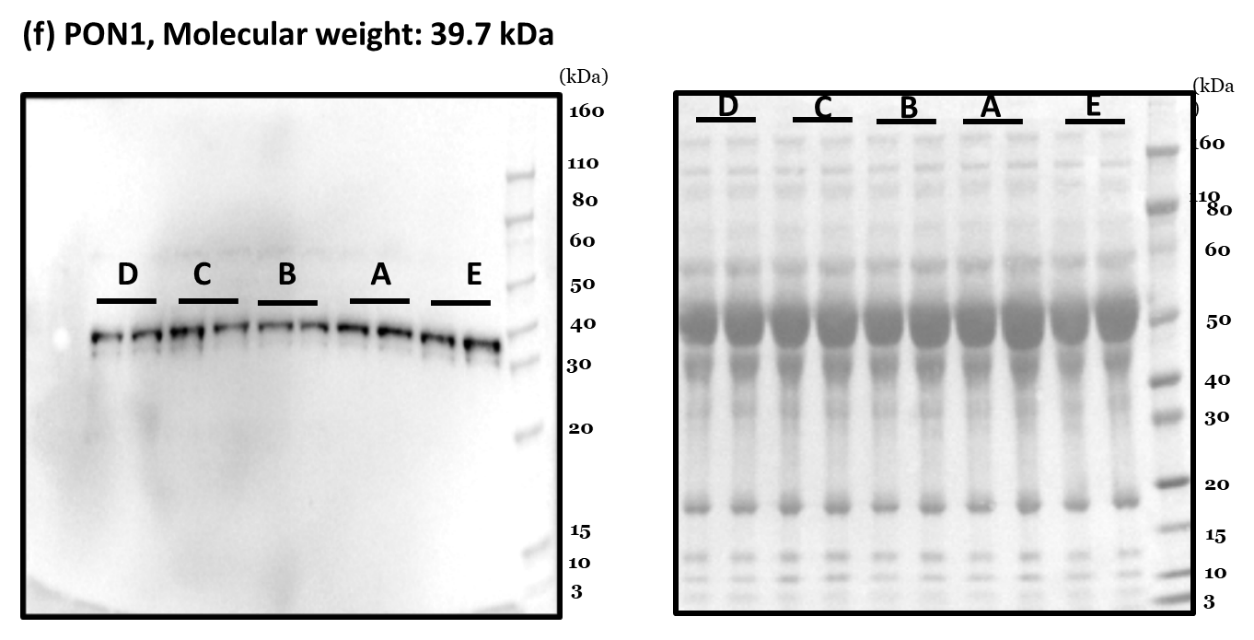
**

**Figure S3**: Western blotting images (left) and Ponceau stained images (right) of **(a)** Complement factor D, CST3, **(b)** Glutathione peroxidase 3, GPX3, **(c)** Complement factor D, CFD, **(d)** Macrophage mannose receptor 1, MRC1, **(e)** Cartilage oligomeric matrix protein, COMP and **(f)** Serum paraoxonase/arylesterase 1, PON1.


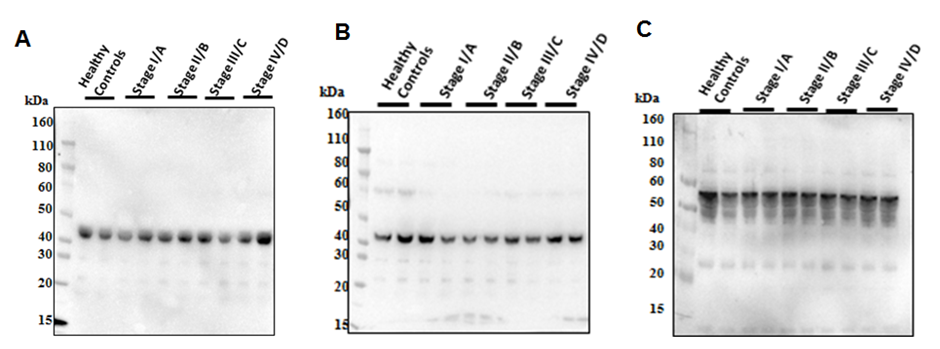


**Figure S4**: Isotype controls for western blots. (a) IgG controls for CST1, MRC1 and COMP (polyclonal Rabbit, IgG). (b) IgG control for CFD and GPX3 (polyclonal goat, IgG). (c) IgG1 control for PON1 (monoclonal rabbit, IgG1).
